# Encapsulated faecal microbiota transfer to target immune activation in patients with cirrhosis and ascites (TransImmune): protocol for a randomised, double-blind, Phase IIa, placebo-controlled trial

**DOI:** 10.1136/bmjopen-2026-119299

**Published:** 2026-08-12

**Authors:** Karsten Große, Frederic Haedge, Carmen Fera, Jana Hecker, Jan Wienstroer, Anastasia Tsakmaklis, Andreas Fichtner, Nicole S Treichel, Thomas Clavel, Theresa Hildegard Wirtz, Oliver Pabst, Rainer Schuckelt, Maria JGT Vehreschild, Tony Bruns

**Affiliations:** 1 Department of Internal Medicine III, University Hospital RWTH Aachen, Aachen, Germany; 2 Center for Translational & Clinical Research Aachen (CTC-A), University Hospital RWTH Aachen, Aachen, Germany; 3 Faculty of Medicine and University Hospital Cologne, University of Cologne, Cologne, Germany; 4 X-Act Cologne Clinical Research GmbH, Cologne, Germany; 5 Functional Microbiome Research Group, Institute of Medical Microbiology, University Hospital of RWTH Aachen, Aachen, Germany; 6 Institute of Molecular Medicine, University Hospital RWTH Aachen, Aachen, Germany; 7 University Hospital Frankfurt, Department II of Internal Medicine, Infectious Diseases, Goethe University Frankfurt, Frankfurt, Germany

**Keywords:** Hepatology, Gastrointestinal Microbiome, Randomized Controlled Trial, Hepatobiliary disease

## Abstract

**Introduction:**

Bacterial translocation and gut dysbiosis are key drivers of systemic immune activation in decompensated cirrhosis, precipitating inflammatory complications such as acute-on-chronic liver failure (ACLF). Currently, no licensed therapies effectively restore intestinal barrier function or reverse dysbiosis in this vulnerable population. While previous studies have suggested benefits of faecal microbiota transfer (FMT) in hepatic encephalopathy or alcohol-associated hepatitis, data on its safety and immunomodulatory effects in decompensated cirrhosis with ascites are lacking. This Phase IIa trial (TransImmune) aims to evaluate the safety and tolerability of encapsulated FMT. Furthermore, it will assess feasibility, microbial engraftment and downstream effects on intestinal barrier integrity, as well as systemic and peritoneal inflammation.

**Methods and analysis:**

This is a prospective, single-centre, randomised, double-blind, placebo-controlled Phase IIa pilot study. A total of 24 patients with decompensated cirrhosis and ascites will be randomised in a 1:1 ratio to receive either encapsulated FMT or placebo over three consecutive days. The investigational product, INTESTIFIX 001, is an encapsulated FMT preparation derived from rigorously screened healthy donors and manufactured under Good Manufacturing Practice (GMP) conditions with predefined release specifications, including minimum alpha-diversity QC criteria, manufactured by the Cologne Microbiota Bank (CMB). The primary endpoints are the occurrence of serious adverse events (SAE) up to the end of study (EOS) and the occurrence and severity of treatment-emergent adverse events (TEAE). Secondary endpoints evaluate signals of clinical efficacy, specifically: (1) systemic inflammation (white blood cell count, C-reactive protein, procalcitonin and IL-6); (2) gut inflammation (faecal calprotectin); (3) organ dysfunction (Child-Pugh, MELD and CLIF-SOFA scores); (4) quality of life (EQ-5D-5L and CLDQ) and (5) the number of antibiotic-free days. Patients will be monitored across five study visits up to 90 days.

**Ethics and dissemination:**

The study was approved by ethics committee review and the German Federal Institute for Drugs and Medical Devices (BfArM). The trial is registered under EU CT no. 2023–5 07 790-18-00. The results of the study will be disseminated via peer-reviewed publications and at international conferences.

**Trial registration number:**

EU Clinical Trials Register: 2023-507790-18-00. Registered on 8 August 2024.

**STRENGTHS AND LIMITATIONS OF THIS STUDY**

- The trial uses a randomised, double-blind, placebo-controlled design, with placebo capsules that are indistinguishable from the active preparation in appearance, weight and taste.
- A GMP-grade faecal microbiota transfer (FMT) preparation (INTESTIFIX 001), manufactured to standardised procedures and predefined release specifications, minimises manufacturing-related and handling-related batch-to-batch variability and allows non-invasive oral administration without endoscopy, while the inherent donor-to-donor batch variability of allogeneic FMT is documented and explored descriptively.
- A multi-compartment sampling approach (blood, stool and ascites) is applied to assess microbial engraftment, intestinal barrier function, and both systemic and peritoneal inflammation.
- As a single-centre pilot study with a small sample size (n=24), the trial is designed to assess safety and feasibility, and its statistical power for clinical efficacy endpoints is very limited.
- Concomitant medications that are often unavoidable in this population (eg, lactulose, proton pump inhibitors and antibiotic prophylaxis) may confound interpretation of microbiome changes, an effect that randomisation distributes across arms but does not eliminate.

## Introduction

 Chronic liver disease represents a major global health burden. In Europe alone, more than 29 million people suffer from chronic liver disease, resulting in approximately 170 000 deaths annually.^1^ While the early stages of the disease are often asymptomatic, the progression to liver cirrhosis marks a critical turning point. Recent data indicate that patients with cirrhosis have the highest in-hospital mortality rate compared with other chronic diseases, with mortality exceeding 17% in those with complicating bacterial infections.^2^ To improve outcomes in this vulnerable population, it is essential to understand the underlying mechanisms that drive the transition from stable, compensated cirrhosis to the acute decompensation phase characterised by organ failure and high mortality.

While the underlying aetiology drives the development of cirrhosis, a key mechanism underlying its progression to decompensation is the dysfunction of the gut-liver axis. Physiologically, the liver acts as a gatekeeper, filtering blood from the intestine and preventing harmful substances from entering the systemic circulation.^3^ In advanced cirrhosis, however, this protective relationship breaks down. The physiological barriers, mucoepithelial, endothelial, and immunological, fail, facilitating the translocation of viable bacteria and microbial products from the intestinal lumen into the portal and systemic circulation.^4^ This process is not merely a consequence of portal hypertension but a key driver that perpetuates systemic inflammation, exacerbates portal hypertension and accelerates the development of acute-on-chronic liver failure (ACLF).^5 6^

Barrier failures in cirrhosis are linked to alterations in the intestinal ecosystem known as gut dysbiosis.^3 7^ Patients with cirrhosis exhibit quantitative and qualitative changes in their microbiota, including small bowel intestinal overgrowth and distinct patterns such as the enrichment of oral commensals (eg, *Streptococcus* and *Veillonella* species) alongside an increase in potentially pathogenic *Enterobacterales*.^8^ This altered microbiota is mechanistically implicated in the progression of metabolic dysfunction-associated steatotic liver disease (MASLD) and alcohol-related liver disease (ALD). Elevated luminal concentrations of ethanol, acetaldehyde and acetate, combined with depleted levels of barrier-protective butyrate and long-chain fatty acids correlate with disrupted tight junction proteins and decreased luminal antimicrobial peptides.^9^

In a healthy state, bacteria that breach the gut barrier and enter portal circulation are efficiently captured and cleared by hepatic Kupffer cells. However, in cirrhosis, this clearance capacity is significantly impaired.^10 11^ Consequently, bacterial products can enter the systemic circulation and then inflammation intensifies and circulating immune cells, such as monocytes, develop a dysfunctional state of altered innate immune memory and immune deficiency.^12–14^ These inflammatory events perpetuate a vicious cycle of translocation and systemic immune activation, significantly increasing the risk of bacterial infections.

Currently, therapeutic options to break this cycle are limited. No licensed therapies effectively reverse dysbiosis or strengthen intestinal barrier function in patients with decompensated cirrhosis and ascites. The management of ACLF remains largely supportive, targeting organ failure rather than the underlying microbial drivers.^15^ Faecal microbiota transfer (FMT) has emerged as a promising strategy to restore microbial diversity and homeostasis. While FMT is established for *Clostridioides difficile* infection,^16^ its application in liver disease is under active clinical evaluation, with several randomised trials now reporting preliminary efficacy data.

Small, controlled trials have demonstrated potential benefits of FMT in MASLD (improving insulin sensitivity and permeability),^17–19^ hepatic encephalopathy (HE; improving cognition, dysbiosis and quality of life)^20–22^ and alcohol-associated hepatitis (improving ACLF and potential survival).^23–26^ Concurrently, large ongoing trials like the UK PROMISE trial (ISRCTN17863382) and the Danish ChiFT trial (NCT04932577)^27^ are evaluating clinical endpoints in broader populations with cirrhosis.

### Rationale for the TransImmune trial

Despite these advances, a critical evidence gap remains regarding patients with cirrhosis and ascites. The presence of ascites is a major predictor of intestinal permeability and poor infection-free survival.^28^ Patients with ascites exhibit the most severe gut barrier dysfunction and the highest infection risk, yet remain systematically under-represented in FMT trials, which have largely enrolled stable outpatient cohorts—a gap the TransImmune trial is specifically designed to address.

Establishing the safety profile in this specific cohort is paramount. Currently, the majority of FMT safety data is derived from patients without liver disease (eg, CDI), where adverse events (AEs) occur in approximately 19% of procedures (mostly mild diarrhoea or abdominal pain), with serious adverse events (SAEs) remaining rare at 1.4%.^29^ Regarding immunocompromised patients, current literature generally supports FMT application due to an acceptable risk profile,^30^ yet severe outcomes, including treatment-related infections, colectomies and death, have been observed in isolated cases among severely immunosuppressed patients.^31^

In patients with cirrhosis, the safety landscape is more nuanced. In a multicentre cohort of patients with cirrhosis undergoing FMT for CDI, FMT-related AEs occurred in 30.2% (mostly self-limited abdominal cramping or diarrhoea) and FMT-related SAEs in 7.9%.^32^ A systematic review of case series has identified severe infectious complications, including *Escherichia coli* bacteraemia and spontaneous bacterial peritonitis, occurring shortly after FMT.^33^ In contrast, prospective randomised trials in cirrhotic cohorts have demonstrated a favourable safety profile. Prospective trials using both enema and capsule formulations reported no increase in SAEs compared with standard care and even a reduction in hospitalisations and alcohol craving in specific subgroups.^21 22 34^ Most recently, the Phase II THEMATIC trial confirmed this trend, reporting no FMT-related SAEs among 60 patients with cirrhosis and HE.^20^ These trials, however, largely excluded patients with advanced organ dysfunction and ACLF, and safety data cannot be directly extrapolated to patients with ascites and profound immune dysfunction, who are at the highest risk for bacterial translocation and infection.

Furthermore, the potential transmission of multidrug-resistant organisms (MDRO) represents a critical safety concern that necessitates rigorous donor screening protocols.^35 36^ Although restoration of a healthy microbiome has been shown to reduce antibiotic resistance genes in the gut,^37^ the potential risk of introducing pathogens remains.

To address these challenges, we use INTESTIFIX 001, a standardised, encapsulated FMT preparation derived from healthy donors with high alpha-diversity, manufactured by the Cologne Microbiota Bank (CMB). This Phase IIa pilot study aims to evaluate the safety and tolerability of this standardised intervention in patients with decompensated cirrhosis and ascites. Furthermore, it will assess feasibility, microbial engraftment and signals of efficacy regarding intestinal barrier integrity, as well as systemic and peritoneal inflammation.

## Methods and analysis

### Study design

TransImmune is an investigator-initiated, prospective, single-centre, randomised, placebo-controlled, double-blind phase IIa trial conducted at the University Hospital RWTH Aachen, Germany, to investigate the safety and tolerability of the FMT product INTESTIFIX 001 in subjects aged 18 to 70 years with decompensated liver cirrhosis and ascites. The study will recruit 24 patients with confirmed cirrhosis and ascites requiring paracentesis or diagnostic tap. The trial is designed in a parallel-group design with two arms randomised in a 1:1 ratio (12 patients INTESTIFIX 001, 12 patients placebo).

### Objectives

The *primary objective* is to evaluate the safety of faecal microbiota transfer (FMT) capsules for the treatment of immune activation in patients with cirrhosis and ascites. The primary endpoints are:

1. The occurrence of serious adverse events (SAEs) recorded at any time during the study, from inclusion until the end of study (EOS) visit.
2. The occurrence and severity of treatment-emergent adverse events (TEAE) and adverse events (AE).

The *secondary objective* is to evaluate signals of clinical efficacy regarding immune activation. Endpoints to assess the secondary objective are as follows:

1. Systemic inflammation: Differences in white blood cell count, C-reactive protein, procalcitonin and IL-6 between baseline (day 0) and V3 (day 7±2), V4 (day 28±3), and EOS (day 90+14).
2. Gut inflammation: Differences in faecal calprotectin between baseline (day 0) and V3 (day 7±2), V4 (day 28±3), and EOS (day 90+14).
3. Organ dysfunction: Differences in Child-Pugh score, model of end-stage liver disease (MELD) score, chronic liver failure-sequential organ failure assessment (CLIF-SOFA / CLIF-C OF) score between baseline (day 0) and V3 (day 7±2), V4 (day 28±3), and EOS (day 90+14).
4. Quality of life: Differences in EQ-5D-5L and CLDQ score between baseline (day 0) and EOS (day 90+14).
5. Antibiotic-free days: Number of days without antibiotic use (except for oral use of norfloxacin or rifaximin) between baseline (day 0) and EOS (day 90+14).

The *exploratory objectives* of this trial aim to evaluate the effectiveness of strain engraftment from the FMT products by shotgun metagenomic sequencing, the longevity of the graft and how these factors affect the intestinal barrier function, as well as systemic and peritoneal inflammation. Exploratory analyses include but are not limited to serum and ascitic fluid markers of immune cell activation, systemic inflammation, acute-phase response and immune activation; the composition of the faecal microbiome, virome and mycobiome by shotgun metagenomic sequencing; circulating and faecal markers of intestinal permeability, colonic inflammation and bacterial translocation; and the frequency, differentiation, cytokine repertoire and cytotoxicity of immune cell subsets in blood and, where clinically indicated, in ascitic fluid—each assessed longitudinally from baseline (day 0) through day 7 (±2), day 28 (±3), and end of study (day 90±14).

### Inclusion/exclusion criteria

#### Key inclusion criteria

- Age 18 to 70 years.
- Confirmed liver cirrhosis with ascites requiring therapeutic paracentesis or a diagnostic ascitic tap within 30 days prior to inclusion.

#### Key exclusion criteria

- Severe acute-on-chronic liver failure (ACLF) grade ≥2 or MELD score >20.
- Active bacterial infection or use of systemic antibiotics within 7 days prior to inclusion (exception: stable prophylaxis with norfloxacin or rifaximin).
- Current treatment with immunomodulatory drugs

Detailed inclusion/exclusion criteria are listed in box 1.

Box 1 **Inclusion and exclusion criteria**

**Inclusion criteria**

1. Subject has cirrhosis of any aetiology based on clinical standard criteria, imaging findings and/or histology.
2. Subject is diagnosed with ascites and has undergone paracentesis or diagnostic ascitic tap within the last 30 days prior to study inclusion.
3. Subject age is between 18 and 70 years at the time of study inclusion.
4. Subject is able to swallow the investigational medicinal product (IMP) faecal microbiota transfer capsules or placebo.
5. Subject is mentally and physically able to understand the significance and scope of the study and to comply with the study protocol.
6. A written informed consent form has been signed prior to participation in the study.

**Exclusion criteria**

1. Subject has acute-on-chronic liver failure (ACLF) grade two or higher according to the EASL-CLIF Consortium definition.
2. Subject has model for end-stage liver disease (MELD) score >20.
3. Subject has received systemic antibiotics within 7 days prior to study inclusion, except for stable doses of norfloxacin or rifaximin.
4. Subject has active bacterial infection at inclusion.
5. Subject has known primary immunodeficiency (eg, Bruton disease or common variable immunodeficiency) or secondary immunodeficiency (eg, HIV infection).
6. Subject has active malignancy or history of malignancy, which was not curatively treated (excluding non-melanoma skin cancer).
7. Subject receives treatment with immunomodulatory drugs (prednisolone, prednisone, methylprednisolone, hydrocortisone, dexamethasone, azathioprine, mercaptopurine, tacrolimus, mycophenolate, cyclosporine, sirolimus, everolimus, hydroxychloroquine, leflunomide, methotrexate, infliximab, adalimumab, etanercept, rituximab, anakinra, tofacitinib, upadacitinib, natalizumab and basiliximab), currently or within 90 days prior to study inclusion.
8. Subject has a history of hypersensitivity or allergies to the investigational capsule coating substances or to any compound of INTESTIFIX 001 or placebo.
9. Subject has an expected lack of compliance.
10. Subject has a life expectancy of less than 6 months.
11. Subject is pregnant or breastfeeding.
12. Subject is not willing to take adequately safe contraceptive measures.
13. Study participation is, at the discretion of the investigator, an unacceptable risk due to pre-existing or concomitant disease or due to the patient’s general underlying condition.
14. There is a current or past medically relevant disease or treatment that could influence the evaluation of the study.
15. Subject has received an investigational drug in another study within the last 30 days before study inclusion.
16. Concurrent participation in another clinical intervention study.
17. Subject is institutionalised due to an official or court order.
18. Subject is in a dependent relationship or employment relationship with the sponsor or investigator.

Alcohol consumption is not an exclusion criterion and is not prohibited during the outpatient phase of the study; however, abstinence is encouraged, and alcohol use is not permitted during inpatient stays in accordance with ward regulations.

### Sample size

As an exploratory Phase IIa pilot trial, the primary purpose is to estimate effect sizes and event rates rather than to perform confirmatory hypothesis testing. Therefore, the width of the 95% CI for the difference in event rates was used as the criterion for sample size calculation. A sample size of 10 evaluable subjects per arm will result in a CI width for the difference of two rates between 0.74 and 0.82 (assuming equal proportions) and ranging between 0.2 and 0.5 using the score method of Miettinen and Nurminen.^38^ Furthermore, 10 subjects per arm ensure that adverse events occurring with a frequency of more than 15% will be detected with a probability of 80%. To account for a potential drop-out rate of approximately 17% (including premature withdrawal and serious protocol violations), a total of 24 participants (12 per arm) will be randomised. Drop-outs will not be replaced.

### Recruitment and randomisation

Eligible patients are approached by investigators at the Department of Internal Medicine III, University Hospital RWTH Aachen. All screened subjects receive a screening number. Screening failures are defined as subjects who consent to participate in the clinical trial but are subsequently not included; their demographic data and the reason for exclusion will be documented. Eligible subjects will be allocated to the intervention (INTESTIFIX 001) or placebo group in a 1:1 ratio at random, using a GCP-compliant web-based tool (www.randomizer.at; Medical University of Graz, Austria). Randomisation will be performed using a permuted block design. As this is a single-centre study, randomisation will not be stratified.

### Blinding

Study medication with the investigational IMP (INTESTIFIX 001 or placebo) is blinded to subjects, investigators and site personnel. The placebo capsules are visually, physically and organoleptically indistinguishable from the verum capsules. The blinding of treatment allocation will be maintained for the sponsor, study site and participants involved in this study until the database is locked. Unblinding may be conducted by the investigator in the event of a medical emergency when it is deemed necessary for the safety and medical treatment of the patient.

### FMT preparation

INTESTIFIX 001 is an encapsulated FMT preparation for oral administration manufactured by the Cologne Microbiota Bank (CMB) in a GMP-certified laboratory. The product is derived from stool donations of healthy donors. Approximately 50 g of donor stool are homogenised with 250 mL of 0.9% saline solution, filtered and processed via centrifugation. The pellet is resuspended with saline and glycerol (final concentration 10%) serving as a cryoprotectant. The resulting suspension is aliquoted into capsules, with each capsule containing a bacterial extract from approximately 1.67 g of donor stool. To ensure colonic release, a double-encapsulation technology is employed, where an inner acid-resistant capsule (Capsugel Enprotect) is placed inside an outer delayed-release capsule (Capsugel DR). Placebo capsules contain 10% glycerol in normal saline and are indistinguishable from INTESTIFIX 001 in appearance, weight and taste.

To account for incubation periods of transmissible infections, a 6 week donation pause is instituted after a 6 week donation period, followed by mandatory re-screening of donor blood. INTESTIFIX 001 capsules are made exclusively from stool samples that test negative for antibiotic-resistant or potentially pathogenic microorganisms. Donors are regularly evaluated for risk factors associated with carrying antibiotic-resistant bacteria, such as recent antibiotic use, healthcare facility visits or travel activities, to ensure a strict screening process that minimises the risk of transmitting infectious agents. Faecal material from different donors is not pooled; each treatment is manufactured from the material (approximately 50 g) derived from one donation of one single qualified donor. Donor and batch identity will be documented and considered in exploratory analyses. Finished capsules are kept in quarantine and are only released for use on confirmation of negative re-screening results. Quality controls include examination of fresh stool from each donation for potentially infectious pathogens and microbiome analysis by 16S rRNA gene amplicon analysis to verify compliance with predefined minimum alpha-diversity release specifications, defined as a Shannon Diversity Index >2 and Richness >30 genera. These IMPD-defined QC criteria are intended to confirm a minimum level of microbial community complexity and to exclude markedly low-diversity donations; they are not validated clinical efficacy thresholds. Additionally, disintegration time of the FMT capsules and fluorescence-based cell counting of enzymatically active cells (> 1 ×10^10^ cells/mL) is conducted as quality control of each batch. The capsules are stored at controlled temperatures ranging from −70 °C to −90 °C. To assure an uninterrupted cold chain, shipment from the manufacturer to the study site is conducted on dry ice with continuous temperature logging.

### Intervention

The treatment regimen consists of a single daily dose of eight capsules, taken together at one time point, on three consecutive days. This results in a total cumulative dose of 24 capsules, corresponding to approximately 40 g of original stool suspension. This dose was selected to optimise the probability of microbial engraftment and barrier restoration given the severe gut-vascular barrier dysfunction inherent to decompensated cirrhosis with ascites, while maintaining acceptable oral tolerability. The IMP, whether INTESTIFIX 001 or placebo capsules, will be taken under the supervision of study personnel at the study site with clear water after a fasting period of at least 2 hours. The IMP is removed from ultra-low temperature storage 10 to 30 min prior to administration to allow the capsules to reach a suitable temperature for intake.

As a specific safety precaution, the first three randomised patients will be dosed sequentially, with each patient observed throughout the 3 day treatment phase before the next patient is dosed. Recruitment of the remaining cohort will proceed without restrictions if no IMP-related serious adverse reactions (SARs) are recorded during the treatment phase of these sentinel participants; if an IMP-related SAR occurs, the subsequent three patients are likewise dosed sequentially. Throughout the study, patients continue to receive standard-of-care treatment. However, IMP administration must be permanently discontinued if a subject develops acute-on-chronic liver failure (ACLF) grade ≥2, a MELD score >20, loses the ability to swallow capsules, requires parenteral antimicrobial therapy for infection (excluding single-shot prophylaxis) or receives immunomodulatory therapy (eg, corticosteroids, calcineurin inhibitors or biologics) during the treatment phase.

### Follow-up

**Figure 1:**
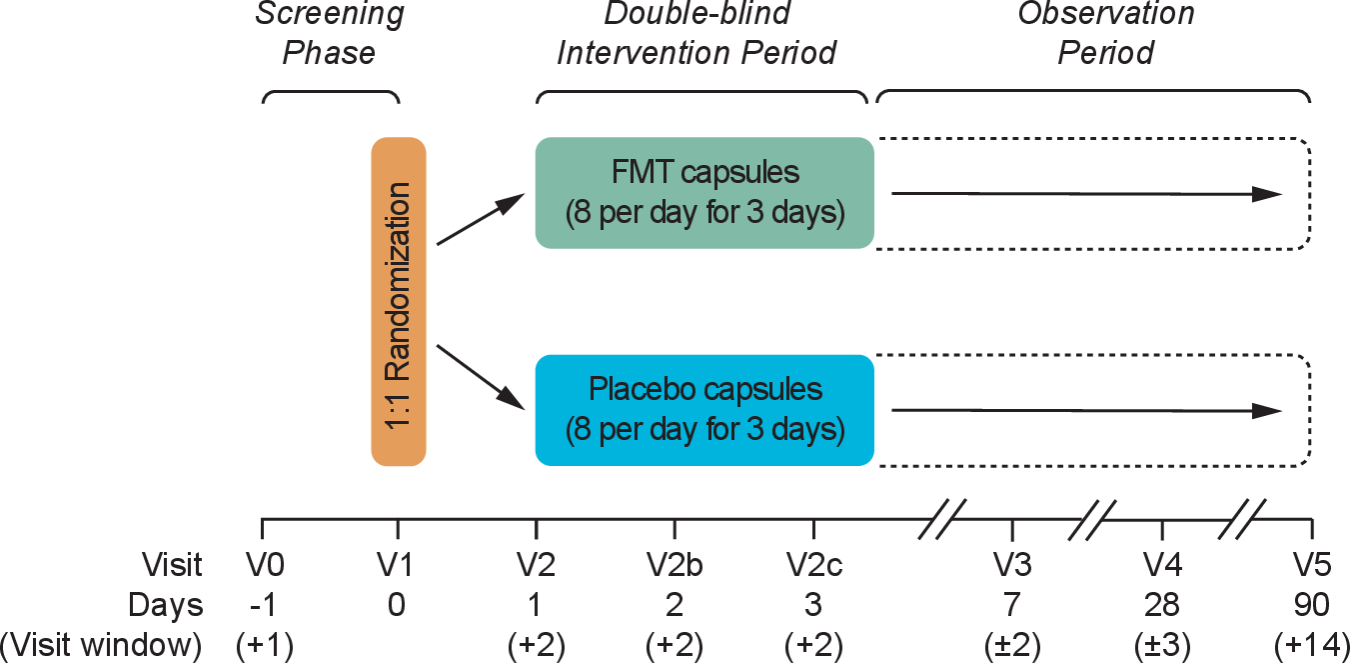
Patient flow chart A total of 24 patients with decompensated cirrhosis and ascites will be randomised in a 1:1 ratio to receive either encapsulated faecal microbiota transfer (FMT, INTESTIFIX 001) or placebo. Following screening (**V0**) and baseline assessment (**V1**), patients will enter a double-blind intervention period receiving eight capsules daily on three consecutive days (V2, V2b, V2c). The observation period includes follow-up visits at day 7 (**V3**), day 28 (**V4**) and day 90 (**V5**). Clinical data, safety parameters and biological samples (blood, stool) will be collected to assess inflammatory markers, immune activation and microbial engraftment by shotgun sequencing. Ascitic fluid will be collected when clinically indicated diagnostic puncture/paracentesis is performed.

Following the screening phase (V0) and the baseline/randomisation visit (V1), the treatment phase spans 3 days (Visits 2, 2b and 2 c), during which the IMP is administered and acute tolerability is monitored. The subsequent follow-up phase includes an end-of-treatment visit at day 7 (±2 days, V3), a follow-up visit at day 28 (±3 days, V4) and the end-of-study visit at day 90 (+14 days, V5) (figure 1).

At each visit, assessments include a complete physical examination, vital signs and a specific clinical assessment for new infections. Safety monitoring involves the continuous recording of adverse events (AEs) and concomitant medications. To evaluate mechanistic endpoints and signals of efficacy, blood and stool samples are collected at baseline, day 7, day 28 and day 90. Quality of life is assessed using the EQ-5D-5L and chronic liver disease questionnaire (CLDQ) at baseline and end of study. Diagnostic ascitic tap or a paracentesis are not study-mandated procedures, and ascitic fluid will be collected when a clinically indicated diagnostic puncture/paracentesis is performed.

### Discontinuation and early termination

Participants may withdraw their consent at any time without providing a reason and without prejudice to their future medical care. An investigator may also withdraw a participant if it is deemed in their best medical interest or if specific discontinuation criteria (eg, pregnancy and major protocol deviation) are met. Participants who discontinue the IMP but do not withdraw consent remain in the study for safety follow-up. The entire study may be terminated prematurely by the sponsor, the competent authority, or the ethics committee if the benefit-risk assessment changes significantly, if safety concerns arise (eg, on recommendation by the data and safety monitoring board) or if the study proves not to be feasible.

### Audits and inspections

Audits will be conducted according to the audit plan for this study. This includes the possibility that a member of the sponsor’s quality assurance unit or a person authorised by the sponsor (auditor) visits the study site on site to check the study conduct. Similarly, all study documents originating from the study site may be audited. Auditors carry out their work independently of studies. In the case of a regulatory audit (inspection), the auditor will be accompanied by a monitor or other authorised person from the sponsor. The sponsor and all participating study sites are obliged to support auditors and inspectors of competent authorities and, in this context, to grant the persons commissioned access to the original documents.

### Safety

Safety monitoring is the primary objective of this trial. Adverse events (AEs) are recorded from the time of informed consent until the end of the study at day 90 (+14 days, V5). The severity of AEs is graded according to the National Cancer Institute Common Terminology Criteria for Adverse Events (NCI-CTCAE) V. 5.0. Any serious adverse event (SAE) must be reported to the sponsor within 24 hours of awareness. The sponsor is responsible for reporting Suspected Unexpected Serious Adverse Reactions (SUSARs) to the EudraVigilance database and relevant ethics committees in accordance with the Clinical Trials Regulation (EU) No 536/2014. An independent data and safety monitoring board (DSMB), comprising two clinicians and an independent biostatistician, continuously monitors participant safety throughout the trial; the schedule and criteria for its safety reviews are pre-specified in the DSMB Charter in addition to ad hoc reviews triggered by any safety concern.

### Data protection, collection and management

All study data will be recorded in a validated electronic Case Report Form (eCRF, Libre Clinica) hosted on access-protected servers. To ensure confidentiality, data are pseudonymised using a unique patient identification number. Access to the database is restricted to authorised personnel via personal accounts with two-factor authentication. The identification list linking patients to their data is stored exclusively at the study site under GCP-compliant conditions and is never copied. Biological samples are stored pseudonymously; traceability is maintained via the site-specific identification list to allow for destruction in case of consent withdrawal. Access to original identifying data is granted solely to authorised persons (eg, monitors, auditors and authorities) for verification purposes. In the event of a data protection violation, the study site must immediately report the breach to the sponsor’s data protection officer for coordinated action. All essential documents will be archived for at least 25 years.

### Statistical analysis

The intention-to-treat (ITT) population includes all randomised patients and will be used for efficacy signals, while the safety (SAF) population includes all randomised patients who took at least one capsule of the IMP and will be used for the primary safety analysis. For the primary endpoint, the percentage of participants experiencing at least one treatment-emergent serious adverse event (SAE) in the INTESTIFIX 001 group will be compared with the placebo group within the safety population up to the end of the study. Given the exploratory nature of this Phase IIa pilot trial, descriptive statistical methods will be employed, and all inferential statistics will be interpreted as hypothesis-generating rather than confirmatory. This approach facilitates the preliminary assessment of efficacy signals and provides data to inform the design of future confirmatory trials.

### Study timeline

The study is scheduled to commence recruitment with the First Patient First Visit (FPFV) in the third quarter of 2025. Recruitment is expected to conclude with the last patient first visit in the fourth quarter of 2026. The last patient last visit (LPLV) is anticipated in the first quarter of 2027. Database lock is planned for the second quarter of 2027, with the final clinical study report (CSR) expected to be completed by the first quarter of 2028.

## Ethics and dissemination

The study was approved by the Ethics Committee of Ulm University as the reporting Member State ethics committee appointed by the German Federal Institute for Drugs and Medical Devices (BfArM) (Reference number: B_02195). The trial is registered under EU CT no. 2023–5 07 790-18-00. The study will be conducted in full accordance with the Declaration of Helsinki, the Declaration of Taipei, the ICH-GCP guidelines and the German Medicines Act (AMG). All participants must provide written informed consent prior to any study-specific procedures (see online supplemental file 1). In accordance with the AMG, the sponsor has concluded a proband insurance policy; additionally, patients are covered by an accident insurance policy for the duration of their participation.

The results of the clinical trial will be disseminated via publication in international peer-reviewed scientific journals and presentations at national and international congresses. For all publications, data protection regulations applicable in the EU will be strictly observed to ensure that no conclusions can be drawn about individual participants. De-identified individual participant data (IPD) that support the findings of this study will be available on reasonable request. Due to the small sample size and potential risk of participant re-identification, data will not be deposited in an open public repository. Access will be restricted to qualified academic researchers for specified research purposes only, subject to a formal protocol evaluation and a signed Data Use Agreement. All requests should be directed to the principal investigator (TB), who acts as the contact person for data access enquiries.

### Sponsor

University Hospital Aachen, AöR (institution under public law). Dean of the Medical Faculty of the RWTH Aachen for the Managing Board of the University Hospital Aachen.

### Latest protocol version

V. 2.0 as of 25.11.2025. Further trial information under https://euclinicaltrials.eu/search-for-clinical-trials/?lang=en&EUCT=2023-507790-18-00.

## Supplementary material

10.1136/bmjopen-2026-119299 online supplemental file 1
