# Supplementary material for: Encapsulated faecal microbiota transfer to target immune activation in patients with cirrhosis and ascites (TransImmune): protocol for a randomised, double-blind, Phase IIa, placebo-controlled trial
Source: BMJ Open. 2026 Aug 12;16(8):e119299. doi: 10.1136/bmjopen-2026-119299 (PMC13475211; doi:10.1136/bmjopen-2026-119299)
Supplement: Supplementary file 1 [file bmjopen-16-8-s001.pdf]

Titel der Klinischen Prüfung:
Verkapselter fäkaler Mikrobiota-Transfer zur Behandlung der Immunaktivierung bei Patienten mit Zirrhose und Aszites (TransImmune) - Eine randomisierte, doppelblinde, placebokontrollierte klinische Pilotstudie der Phase IIa

**Prüfzentrum**	**Uniklinik RWTH Aachen**<br>Medizinische Klinik III<br>Pauwelsstr. 30<br>52074 Aachen
**Hauptprüfer**	**Univ.-Prof. Dr. med. Tony Bruns**<br>Tel: [redacted]<br>[redacted]
**Coordinating Investigator**	**Univ.-Prof. Dr. med. Tony Bruns**<br>Tel: [redacted]<br>[redacted]
**Sponsor der klinischen Prüfung**	**Universitätsklinikum Aachen, AöR**<br>[redacted]
**Studiennummer des Sponsors**	[redacted]
**EU CT- Nummer**	2023-507790-18

Sehr geehrte Patientin, sehr geehrter Patient,

wir möchten Sie fragen, ob Sie bereit sind, an der nachfolgend beschriebenen klinischen Prüfung (Studie) teilzunehmen.

Klinische Prüfungen sind notwendig, um Erkenntnisse über die Wirksamkeit und Verträglichkeit von Arzneimitteln zu gewinnen oder zu erweitern. Deshalb schreibt der Gesetzgeber im Arzneimittelgesetz vor, dass neue Arzneimittel klinisch geprüft werden müssen.

Die klinische Prüfung, die wir Ihnen hier vorstellen, wurde – wie es das Gesetz verlangt – von der zuständigen nationalen Behörde unter Einbeziehung der national zuständigen Ethikkommission genehmigt.

Diese klinische Prüfung wird in Aachen durchgeführt; es sollen insgesamt ungefähr 24 Personen daran teilnehmen. Die klinische Prüfung wird veranlasst und finanziert durch die Medizinische Fakultät und Eigenmittel der Klinik für Gastroenterologie, Stoffwechselerkrankungen und Internistische Intensivmedizin, organisiert durch den Sponsor Universitätsklinikum Aachen, Anstalt des öffentlichen Rechts (AöR).

Ihre Teilnahme an dieser klinischen Prüfung ist freiwillig. Sie werden in diese Prüfung also nur dann einbezogen, wenn Sie dazu schriftlich Ihre Einwilligung erklären. Sofern Sie nicht an der klinischen Prüfung teilnehmen oder später aus ihr ausscheiden möchten, entstehen Ihnen daraus keine Nachteile.
Auch ist es nur möglich, dass Sie an dieser Prüfung teilnehmen, wenn Sie der Sammlung von Biomaterialien (Blut, Stuhl, ggf. Bauchwasser) zustimmen.

Der nachfolgende Text soll Ihnen die Ziele und den Ablauf erläutern. Der Text ist in drei Teile gegliedert:

- Kurzdarstellung der klinischen Prüfung
- Teil I: Informationen zum Ablauf der klinischen Prüfung einschließlich der damit zusammenhängenden gesundheitlichen Gesichtspunkte
- Teil II: spezifische Informationen zum Datenschutz und zu den Biomaterialien (Blut, Stuhl, ggf. Bauchwassser)

Auf jeden Fall wird ein Prüfer ein Aufklärungsgespräch mit Ihnen führen. Bitte zögern Sie nicht, alle Punkte anzusprechen, die Ihnen unklar sind. Sie können gerne Sätze/Abschnitte markieren, die Sie nicht verstanden haben, um sie mit dem aufklärenden Prüfer zu besprechen. Sie werden danach ausreichend Bedenkzeit erhalten, um über Ihre Teilnahme zu entscheiden.

Kurzdarstellung der klinischen Prüfung

Grund für die klinische Prüfung: Sie sind an einer Lebererkrankung Leberzirrhose mit Aszites erkrankt und Sie leiden an Komplikationen. Typisch für diese Erkrankung sind eine gestörte Darmbarriere und eine veränderte Zusammensetzung der Darmmikrobiota. Als Darmmikrobiota bezeichnet man die Gesamtheit aller Mikroorganismen (z.B. Bakterien, Viren und Pilze), die den Darm besiedeln. Durch die Lebererkrankung werden die Darmwände durchlässig und es treten Bestandteile des Darminhalts und Bakterien in den Blutkreislauf über und verursachen Entzündungsprozesse, Infektionen und andere Komplikationen der Leberzirrhose. Zurzeit gibt es keine zugelassene Behandlung, welche die Funktion der Darmbarriere bei Leberzirrhose wirksam verbessern kann. Die Wiederherstellung einer gesunden Darmmikrobiota kann zu einer verbesserten Darmbarriere beitragen.

Das Prüfpräparat mit dem Namen INTESTIFIX 001 enthält verkapselte fäkale Mikrobiota (aufgereinigte Stuhlbestandteile) von gesunden Spender(n) und wird in dieser klinischen Prüfung getestet, um herauszufinden, ob es sicher und verträglich ist.

Das getestete Prüfpräparat: INTESTIFIX 001 ist noch in der klinischen Prüfung und wurde für Ihre Behandlung bisher nicht zugelassen.

Ablauf der klinischen Prüfung: Wenn Sie sich zur Teilnahme entscheiden, dauert die klinische Prüfung für Sie 90 Tage. In dieser Zeit werden Sie mindestens acht Termine in der Prüfstelle wahrnehmen. Diese dauern etwa 30 Minuten, wobei die Dauer der Termine an denen Sie Prüfpräparate einnehmen davon abhängt wie gut und schnell Sie die Kapseln schlucken können.

Zunächst wird untersucht, ob Sie für die Teilnahme an dieser klinischen Prüfung geeignet sind. Anschließend werden zwei zufällige Patientengruppen gebildet. Die Zuordnung zu den Behandlungsgruppen erfolgt nach dem Zufallsprinzip (randomisiert).

Dabei werden Patienten in der einen Gruppe für drei Tage INTESTIFIX 001-Kapseln einnehmen, während die andere Gruppe ein Placebo erhält. Bei dem Placebo handelt es sich um Kapseln ohne Wirkstoff, welche äußerlich nicht von den Kapseln mit Wirkstoff unterschieden werden können. Weder Ihr Prüfer noch Sie wissen also, ob Sie das Prüfpräparat mit oder ohne Wirkstoff erhalten. Dabei spricht man auch von einer doppelten Verblindung.

7 (±2), 28 (±3) und 90 (+14) Tage nach der letzten Einnahme werden Sie erneut vom Prüfer untersucht. Nach der letzten Untersuchung werden Sie dann nach den gültigen Behandlungsstandards weiterbehandelt.

Bei allen Untersuchungen werden Sie zu Ihrer Krankheit befragt. Außerdem werden Sie körperlich untersucht, Ihnen wird Blut abgenommen und Sie erhalten ein Entnahmeset, mit dem Sie selbst eine Stuhlprobe entnehmen können. Wann genau welche Untersuchung bzw. Probennahme durchgeführt wird, können Sie dem Ablaufplan (Seite 6) entnehmen.

Eine Beschreibung dieser klinischen Prüfung wird in der europäischen Datenbank für klinische Prüfungen (Clinical Trials Information System – CTIS) unter https://euclinicaltrials.eu/search-clinical-trials-reports/ gemäß EU-Recht (EU CT Nummer 2023-507790-18) verfügbar sein. Diese Website

enthält keine Informationen, die Sie identifizieren können. Die Website enthält höchstens eine Zusammenfassung der Ergebnisse. Sie können diese Website jederzeit durchsuchen.

Möglicher Nutzen für Sie: Aufgrund der bisherigen Forschungsergebnisse erhofft man sich, dass die Behandlung mit INTESTIFIX 001-Kapseln zu einer Normalisierung Ihrer Darmmikrobiota, Verbesserung der Darmbarriere und zur Verringerung von Entzündungsprozessen führt. Das ist bisher jedoch nicht nachgewiesen. Es kann deshalb sein, dass diese Behandlung für Sie keinen Nutzen haben wird.

Risiken und Belastungen: Die Teilnahme an einer klinischen Prüfung ist mit Risiken und Belastungen verbunden:

Das Prüfpräparat kann Nebenwirkungen haben, die über die Risiken Ihrer Standardtherapie hinausgehen. Dazu gehören vor allem Magen-Darm-Nebenwirkungen wie Durchfall und Bauchbeschwerden. Daneben sind leichtere Nebenwirkungen zu erwarten. In seltenen Fällen können aber auch andere schwere Nebenwirkungen auftreten.

Die studienbedingten Untersuchungen sind regelmäßig mit leichteren Beeinträchtigungen verbunden. Darüber hinaus besteht ein Risiko der Übertragung antibiotika-resistenter Bakterien und Infektionen durch den Spender, welches aber durch ein umfassendes mehrstufiges Screening (Auswahlverfahren) des Spendermaterials verringert wird.

Außerdem bestehen Risiken bei der Einnahme der INTESTIFIX 001- oder Placebo-Kapseln, weswegen die Einnahme nur unter Aufsicht von Studienpersonal stattfindet.

Eine ausführlichere Angabe der Risiken und Belastungen finden Sie auf den Seiten 10-11. Befragen Sie den Prüfer ausführlich dazu.

Freiwilligkeit: Es ist Ihre freie Entscheidung, ob Sie an dieser klinischen Prüfung teilnehmen möchten oder nicht. Sie können jederzeit NEIN sagen, sofort oder auch später. Sie brauchen dafür keine Gründe anzugeben und haben keine Nachteile.

Zusätzlich zur schriftlichen Information werden Sie mündlich aufgeklärt. Fragen Sie den Prüfer, wenn Sie etwas nicht verstehen. Sie haben anschließend ausreichend Bedenkzeit, um sich für oder gegen eine Teilnahme zu entscheiden. Wenn Sie sich für die Teilnahme entscheiden, unterschreiben Sie bitte die Einwilligungserklärung.

Widerruf: Auch nach Einwilligung zur Teilnahme haben Sie im Verlauf der Studie jederzeit das Recht diese zu widerrufen. Dies gilt auch für Ihre Einwilligung zur Verarbeitung Ihrer Daten und der Verwendung Ihrer gespendeten Biomaterialien.

Ausführliche Informationen finden Sie auf den Seiten 17 – 20. Sollten Sie etwas nicht verstehen, sprechen Sie bitte Ihren Prüfer darauf an.

Teil I: Informationen zum Ablauf der klinischen Prüfung und zu gesundheitlichen Aspekten

I.1. Warum und mit welchem Prüfpräparat wird diese klinische Prüfung durchgeführt?

Bislang gibt es für PatientInnen mit Leberzirrhose mit Aszites keine zugelassene Behandlung, die die Funktion der Darmbarriere verbessert und Entzündungsprozesse reduziert. Von der Durchführung der vorgesehenen klinischen Prüfung erhoffen wir uns, die Sicherheit und Verträglichkeit des Therapieverfahrens zu untersuchen, um dieses Therapieverfahren in Zukunft einsetzen zu können. Außerdem sollen die Durchführbarkeit, die Wirksamkeit der Übertragung, die Langlebigkeit des übertragenen Materials (Transplantats) und die damit zusammenhängenden Messwerte ermittelt werden.

Bei dem im Rahmen dieser klinischen Prüfung zu testenden Medikament INTESTIFIX 001– nachfolgend als Prüfpräparat bezeichnet – handelt es sich um ein Arzneimittel in klinischer Erprobung, d.h. es ist von der Behörde für die Behandlung Ihrer Krankheit noch nicht zugelassen.

I.2. Erhalte ich das Prüfpräparat auf jeden Fall?

Im Rahmen dieser klinischen Prüfung werden INTESTIFIX 001-Kapseln mit einem Placebo verglichen. Bei einem Placebo handelt es sich um eine identisch aussehende Kapsel, die jedoch keinen Wirkstoff enthält. Im Falle Ihrer Teilnahme an dieser klinischen Prüfung werden Sie entweder INTESTIFIX 001-Kapseln oder das Placebo erhalten. Der Vergleich mit dem Placebo dient dazu, mögliche Nebenwirkungen von INTESTIFIX 001-Kapseln besser beurteilen zu können. Ob Sie das Placebo oder das Prüfpräparat erhalten, entscheidet ein zuvor festgelegtes Zufallsverfahren, vergleichbar mit dem Werfen einer Münze; dieses Verfahren wird Randomisierung genannt. Die Wahrscheinlichkeit, INTESTIFIX 001-Kapseln zu erhalten, beträgt 50 %.

I.3. Wie ist der Ablauf der klinischen Prüfung und was muss ich bei meiner Teilnahme beachten?

Insgesamt dauert die klinische Prüfung für Sie 92 (+ max. 14) Tage. Dabei wird der Prüfer Sie an mindestens acht Terminen behandeln. Für den Erfolg der klinischen Prüfung und für Ihre eigene Sicherheit ist es sehr wichtig, dass Sie an allen Terminen zu den vorgegebenen Zeitpunkten teilnehmen.

Untenstehend finden Sie zunächst eine Übersicht der Termine und anschließend eine Darstellung des Ablaufs der klinischen Prüfung:

**Termin**	**T0** Screening	**T1**	**T2**	**T2b**	**T2c**	**T3** Ende der Behandlung	**T4** Nach-beobachtung	**T5** Ende der Prüfung	**TPx** Parazentese
**Zeitfenster +/- Kalendertage**	Tag -1 (+1 Tag)	Tag 0	Tag 1 (+2 Tage)	1 Tag nach T2	2 Tage nach T2	Tag 7 (±2 Tage)	Tag 28 (±3 Tage)	Tag 90 (+14 Tage)	Innerhalb Routine
**Aufklärung und Studieneinwilligung**	X								
**Körperliche Untersuchung**	X	X	X			X	X	X	
**Einnahme Prüfpräparat am Prüfzentrum**			X	X	X				
**Blutentnahme**	X		X			X	X	X	
**Blutentnahme (Biomarker)**		X	X			X	X	X	X
**Schwangerschaftstest (Urin)**	X							X	
**Bereitstellung Stuhlproben Kit**	X	X	X			X	X	X	X
**Abgabe Stuhlproben**		X	X			X	X	X	X
**Entnahme Bauchwasser**	(X)	(X)				(X)	(X)		X
**Fragebögen**		X						X	

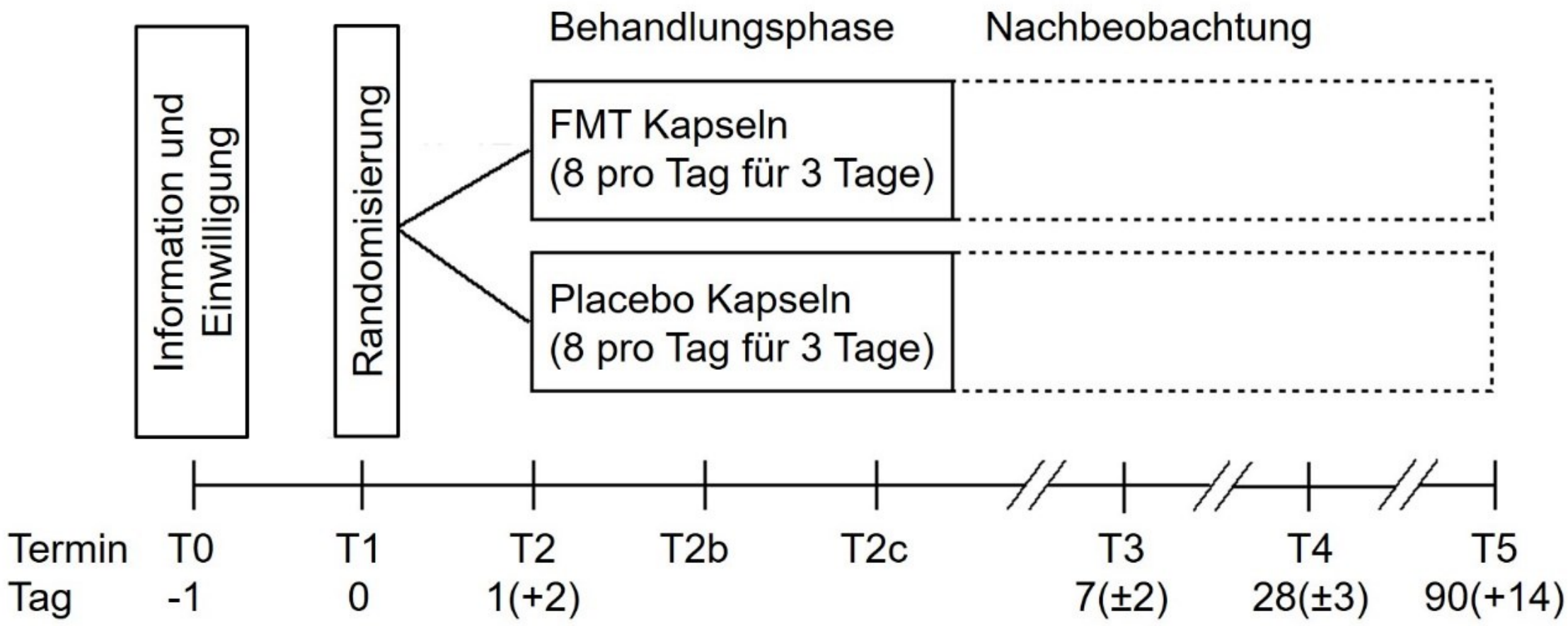

Falls Sie sich für eine Teilnahme an der klinischen Prüfung entscheiden und die Einwilligungserklärung unterzeichnen, wird am ersten Termin (T0) untersucht, ob Sie für die Teilnahme an der Prüfung geeignet sind. Dabei wird die Vorgeschichte Ihrer Krankheit erhoben und Sie werden vom Prüfer untersucht. Dazu gehört insbesondere eine vollständige körperliche Untersuchung, die Entnahme einer Blut- und eine Stuhlprobe. Die Stuhlprobe wird dabei von Ihnen selbst mit Hilfe eines Ihnen zur Verfügung gestelltem Entnahmesets (Kit), genommen. Die durchgeführten Untersuchungen werden weiter unten in diesem Abschnitt genauer dargestellt. Ob Sie an dieser klinischen Prüfung teilnehmen können, hängt von den Ergebnissen dieser Voruntersuchung ab.

Behandlungsphase
Sollten Sie für die Teilnahme geeignet sein, werden Sie am selben oder folgenden Tag erneut untersucht (Termin 1, T1). Hier wird auch erneut überprüft, ob Sie weiter an der Prüfung teilnehmen können. Anschließend erhalten Sie für drei Tage entweder das Prüfpräparat oder Placebo. Da die Kapseln des Prüfpräparats relativ groß sind und jeweils 8 Kapseln eingenommen werden müssen, ist insbesondere wichtig, dass Sie in der Lage sind, diese zu schlucken. An diesen drei Tagen sollten Sie lediglich ein kleines Frühstück zu sich nehmen. Vor der Einnahme der Kapseln sollten Sie 2 Stunden lang fasten. Das Trinken von Wasser ist immer erlaubt. Die Verabreichung der Medikation findet an allen drei Tagen (Termin 2, Termin 2b und Termin 2c) unter Aufsicht des Studienpersonals statt, da diese bei sehr niedrigen Temperaturen gelagert werden müssen und nur sehr kurz vor Einnahme aus dem Gefrierschrank entnommen werden dürfen. Daher ist es nicht möglich das Prüfpräparat mit nach Hause zu nehmen.

Sollte die Behandlung mit dem Prüfpräparat bei Ihnen abgebrochen werden, ist es für Ihre Sicherheit wichtig, dass Sie trotzdem die Studientermine T3, T4 und T5 wahrnehmen.

Ende der Behandlung
Termin 3 findet 7 (±2) Tage nach Termin 0 statt. Hier findet erneut eine Untersuchung durch den Prüfer statt und Sie werden zu Ihrem Befinden befragt.

Nachverfolgung
An Termin 4 nach 28 Tagen (±3 Tage) werden Sie nochmals vom Prüfer untersucht und ebenfalls zu Ihrem Befinden befragt.

Der letzte Termin (T5) findet 90 (+14) Tage nach Termin 0 statt. An diesem Termin werden Sie abschließend durch den Prüfer untersucht. Nach dem Ende der klinischen Prüfung werden Sie nach den gültigen Standards weiterbehandelt.

Zusätzliche Termine
Wird bei Ihnen zwischen Termin 1 und 4 innerhalb der klinischen Routine eine Parazentese (Das Ablassen von Flüssigkeit in Ihrer Bauchhöhle mit einer Nadel zur Linderung der Beschwerden Ihrer Aszites) durchgeführt oder eine diagnostische Punktion (Die Entnahme einer kleinen Menge Bauchflüssigkeit für Diagnosen) vorgenommen, kann dies einen zusätzlichen Studientermin auslösen. Während dieses zusätzlichen Besuchs werden ebenfalls Proben von Biomaterialien wie unten beschrieben gesammelt.

Des Weiteren können Sie selbst um weitere Termine bitten. Sollten sich Ihre Symptome verschlechtern oder Auffälligkeiten auftreten, kann auch Ihr Prüfer einen ungeplanten Termin vorschlagen.

Während der gesamten klinische Prüfung werden folgende Untersuchungen wie in der Übersichtstabelle gezeigt durchgeführt:

- **Anamnese:** Sie werden nach Ihrer Gesundheit sowie nach Krankheiten und Medikamenten gefragt, die Sie einnehmen oder in der letzten Zeit eingenommen haben (einschließlich rezeptfreier Medikamente, Vitamine und pflanzlicher Arzneimittel).
- **Körperliche Untersuchung:** Dies umfasst die Messung Ihrer Vitalfunktionen (Herzfrequenz, Blutdruck, Temperatur, Atemfrequenz, Sauerstoffsättigung im Blut), Größe und Gewicht und Prüfung auf hepatische Enzephalopathie (hierbei handelt es sich um eine Funktionsstörung des Gehirns, die durch eine unzureichende Entgiftungsfunktion der Leber aufgrund Ihrer Lebererkrankung entstehen kann).
- **Klinische Bewertung von Infektionen:** Sie werden im Hinblick auf mögliche Infektionen untersucht.
- **Bewertung Ihres Organversagens** nach verschiedenen standardisierten Kriterien. Dazu werden zum Beispiel anhand Ihrer Laborwerte sogenannte „Scores" (Punktezahl) berechnet, welche eine einheitliche Beschreibung Ihrer Lebererkrankung ermöglichen sollen.
- **Blutuntersuchung für Standard-Laborwerte:** Ihnen wird Blut abgenommen. Pro Termin wird ca. 1 Teelöffel (5 - 6 ml) Blut entnommen. Dabei werden Ihre Blutchemie, Blutfette, Ihr Blutbild und Entzündungswerte überprüft. Sollten bei Ihren Blutwerten Abweichungen festgestellt werden, wird Ihnen möglicherweise an zusätzlichen Terminen erneut Blut abgenommen.
- **Schwangerschaftstests:** Wenn Sie eine Frau sind und Kinder bekommen können, wird bei Ihnen bei Termin 0 (Beginn) und Termin 5 (Ende der klinischen Prüfung) ein Urintest durchgeführt, um festzustellen, ob Sie schwanger sind.
- **Stuhlproben für Standard-Laborwerte:** Sie erhalten ein Entnahmeset (Kit), mit dem Sie zuhause selbst Stuhlproben entnehmen können, welche später im Labor untersucht werden. Das Studienteam wird Ihnen erklären, wie sie das Kit verwenden sollten.
- **Bauchwasser-Proben (siehe Abschnitt zusätzliche Termine):** Diese Probenentnahme wird

nur durchgeführt, sollte bei Ihnen eine diagnostische Aszitespunktion oder eine therapeutische Parazentese durchgeführt werden. Die Proben werden für weitere Forschung zu einem späteren Zeitpunkt gelagert.

- **Fragebögen:** Ihnen werden eine Reihe von Fragen gestellt, die sich auf Ihre allgemeine Lebensqualität und Ihre chronische Lebererkrankung beziehen. Es ist wichtig, dass Sie diese Fragen so vollständig und wahrheitsgemäß wie möglich beantworten. Das Studienpersonal weist Sie an, wie Sie die Fragebögen ausfüllen sollen. Das Beantworten aller Fragebögen kann bis zu 20 Minuten Ihrer Zeit in Anspruch nehmen.
- **Biomarker-Analysen:** Hierzu wird Ihnen Blut abgenommen, Stuhlproben und Proben einer Bauchwasserpunktion gesammelt. Diese Proben werden für Forschung innerhalb dieser klinischen Prüfung gelagert und untersucht. Pro Termin werden ca. 8 Teelöffel (40 ml) Blut entnommen. Für die Stuhlprobe wird ein Teil der von Ihnen für die medizinisch notwendigen Untersuchungen gesammelten Proben abgetrennt. Bauchwasserproben werden nur gesammelt, sofern eine Punktion medizinisch notwendig wurde.
- **Biobank:** Hierfür erhalten Sie eine separate Patienteninformation und Einwilligungserklärung. Eine Zustimmung zur Lagerung und Verwendung Ihrer Biomaterialproben über diese Studie hinaus ist unabhängig von Ihrer Teilnahme an dieser Studie.

Zusätzlich können Daten aus der klinischen Routine für diese klinische Prüfung erhoben werden, sofern sie für diese relevant sind. Das können z.B. Ergebnisse aus bildgebenden Verfahren, Daten zu bakteriellen Infektionen oder zu Ihrem Ernährungszustand sein.

Bitte informieren Sie Ihren Prüfer oder das Studienteam, wenn Sie irgendwelche ungewöhnlichen Symptome haben.
Bei jedem Termin während der klinischen Prüfung werden Sie von Ihrem Prüfer oder einem Mitglied des Studienteams gefragt,

- wie Sie sich fühlen und ob bei Ihnen irgendwelche Nebenwirkungen aufgetreten sind.
- ob Sie im Krankenhaus waren oder eine schwerwiegende Erkrankung hatten.
- welche Medikamente Sie zusätzlich zu Ihrer Studienbehandlung anwenden.

Möglicherweise werden Sie gebeten, während der klinischen Prüfung und nach Abschluss der klinischen Prüfung zum Studienzentrum zurückzukehren, um abweichende Labortests nachzuverfolgen. Das Studienpersonal kann Sie auch anrufen, um ungelöste unerwünschte Ereignisse (Nebenwirkungen) nachzuverfolgen. Für Ihre Sicherheit ist es wichtig, dass Sie den Anforderungen des Studienpersonals nachkommen, zum Studienzentrum zurückzukehren und/oder auf Telefonanrufe zu antworten. Sie können möglicherweise nicht an zukünftigen klinischen Prüfungen teilnehmen, wenn das Studienpersonal nicht sicherstellen kann, dass alle Ihre abweichenden Ergebnisse auf ein akzeptables Niveau zurückgekehrt sind und alle Ihre unerwünschten Ereignisse behoben wurden.

Zusätzliche Medikamente (auch rezeptfreie), von denen der Prüfer noch nichts weiß, dürfen Sie – außer bei Notfällen – nur nach Rücksprache mit Ihrem Prüfer einnehmen. Wenn Sie von anderen Ärzten behandelt werden, müssen Sie diese über Ihre Teilnahme an der klinischen Prüfung informieren. Auch Ihr Prüfer muss über jede medizinische Behandlung, die Sie durch einen anderen Arzt während der Teilnahme an dieser klinischen Prüfung erhalten, informiert werden. Sie erhalten einen Studienausweis, den Sie auch für den Notfall immer mit sich führen sollten.

Alle Medikamente, die Sie im Verlauf dieser klinischen Prüfung bekommen, sollten Sie so sicher aufbewahren, dass sie für Kinder oder andere Personen nicht zugänglich sind. Die Weitergabe an Dritte ist untersagt.

Sie werden selbst Stuhlproben mit Hilfe eines Ihnen zur Verfügung gestelltem Entnahmesets nehmen und bei dem Studienteam abgeben. Bitte beachten Sie dabei die beiliegende Anleitung.

I.4. Welchen persönlichen Nutzen habe ich von meiner Teilnahme an der klinischen Prüfung?

Wenn Sie das Prüfpräparat erhalten, können möglicherweise die Komplikationen Ihrer Begleiterkrankung gelindert werden. Frühere Forschung zu fäkalem Mikrobiota-Transfer (FMT) bei Patienten mit Leberzirrhose haben eine Verbesserung der Zusammensetzung der Darmmikrobiota und der Darmbarriere gezeigt. Bei Menschen, die aufgrund der Lebererkrankung eine Verminderung der Hirnleistung (hepatische Enzephalopathie) aufwiesen, hat ein FMT zur Verbesserung der Aufmerksamkeit und Hirnleistung beigetragen.

Da die Wirksamkeit des Prüfpräparats noch nicht erwiesen ist, ist es jedoch auch möglich, dass Sie durch Ihre Teilnahme an dieser klinischen Prüfung nicht den erhofften Nutzen haben. Einen zusätzlichen Nutzen durch die Teilnahme haben Sie auch dann nicht, wenn Sie nicht das Prüfpräparat erhalten, sondern ein Placebo.

Unabhängig davon, ob Sie einen persönlichen Nutzen von der Teilnahme haben, können die Ergebnisse der klinischen Prüfung möglicherweise dazu beitragen, die Behandlung der Komplikationen von Leberzirrhose mit Aszites zukünftig zu verbessern.

I.5. Welche gesundheitlichen Risiken und Belastungen sind mit meiner Teilnahme an der klinischen Prüfung verbunden?

Die Behandlung mit INTESTIFIX 001-Kapseln kann zu unerwünschten Wirkungen oder Beschwerden führen. Die bislang beobachteten unerwünschten Wirkungen und Beschwerden bei fäkaler Mikrobiota-Übertragung für verschiedene Erkrankungen umfassen, wobei für die meisten noch kein gesicherter Zusammenhang festgestellt wurde:

System-organklasse	sehr häufig	häufig	gelegentlich	Selten	Nicht bekannt
Erkrankungen des Magen-Darmtrakts	Durchfall	Bauchschmerzen, Krämpfe, Unwohlsein. Übelkeit, Erbrechen, Starke Blähungen, Blähbauch, Schub einer chronischen	Stuhldrang, Schmerzen im Enddarm oder am After, Aufstoßen, Rückfluss von Nahrung, Frisches Blut im Stuhl, Blutung aus dem End-darm, Virusbedingte	Verminderter Appetit, Verlust des Appetits, Schleimiger Stuhl, Darmgeräusche, Infektiöse Darm-entzündung, Entzün-dung kleiner Aus-stülpungen im Dick-darm, Divertikulitis,	Blinddarmentzündung, Bluterbrechen, stark erweiterter Dickdarm, toxisches Megakolon, Dünndarmverschluss, Dünndarmdurchbruch, Neue Diagnose einer chronischen Darmentzündung,

		Darmerkrankung, Verstopfung	Dickdarm-entzündung, CMV-Kolitis, Unange-nehmes Gefühl im Bereich des Afters und Enddarms, Kapselreste im Stuhl	spontane bakterielle Bauchfellentzündung	Enddarmvorfall, Bauchspeichel-drüsenentzündung
**Stoffwechsel- und Ernährungsstö rungen**				Gewichtszunahme	Hitzewallungen
**Erkrankungen der Haut**			Hautausschlag, Hautrötung, Juckreiz,	Gürtelrose	Ekzem, Nesselsucht
**Erkrankungen des Nervensystems**			Kopfschmerzen	Schwindel, Taub-heitsgefühl, Kribbeln oder Schmerzen in Armen und Beinen, periphere Neuropathie	Gleichgewichtsstörung-en, Chronische Auto-immunerkrankung, Myasthenia gravis, Depression
**Erkrankungen des Urogenitaltrakt s**					Hodenschmerzen, Akutes Nierenversagen, Harnwegsinfektion
**Erkrankung des Blut- und Immunsystems**					Blutarmut, Mangel an Blutplättchen, Mangel an weißen Blutkörperchen, Immunbedingter Mangel an Blutplättchen, Sjögren-Syndrom Entzündliche Gelenkserkrankung, rheumatoide Arthritis
**Erkrankung der Leber und Galle**					Vorrübergehende Erhöhung der Leberwerte / Bilirubin, Gallensteine
**Erkrankung des Herz—Kreislauf-Systems**					Brustschmerzen, Herzschwäche, Herzrhythmusstörungen, Herz-Kreislaufprobleme, Ereignis im Gehirngefäßsystem, Vorübergehende Kreislaufdepression, autonome Funktionsstörung, Vorhofflimmern
**Erkrankung der Atemwege**			Halsschmerzen	Infektion der oberen Atemwege, Schnupfen,	Verstopfte Nase, Lungenentzündung, Lungenblutung, Allergische Bronchitis

**Allgemeine Erkrankungen/ Beschwerden**		Fieber, Müdigkeit, allgemeines Krankheitsgefühl	Schüttelfrost	Bakterien im Blut, Blutvergiftung, Infektion mit Clostridium perfringens	

Es besteht ein Risiko der Übertragung antibiotika-resistenter Bakterien und Infektionen durch den Spender. Das bedeutet, dass diese Bakterien gegenüber Antibiotika widerstandsfähig sind. Bei einer Infektion bleibt dann das Antibiotikum, gegen das der Erreger resistent ist, wirkungslos. Dieses Risiko wird aber durch ein umfassendes mehrstufiges Auswahlverfahren des Spendermaterials verringert. Auch besteht ein geringes Risiko sich durch die Einnahme der FMT-Kapseln mit einem respiratorischen Virus inkl. SARS-CoV-2 infizieren.

Des Weiteren bestehen Risiken bei der Einnahme der INTESTIFIX 001- oder Placebo-Kapseln. Unzureichendes Schlucken kann zum Eindringen der Kapsel in die Atemwege oder akuten Beschwerden wie Kurzatmigkeit, Erstickung, Ohnmacht oder selbst zum Tod führen. Sollten Sie nicht in der Lage sein, die Kapseln ordnungsgemäß zu schlucken, können Sie nicht (weiter) an der klinischen Prüfung teilnehmen.

Darüber hinaus können die im Rahmen dieser klinischen Prüfung durchgeführten Maßnahmen mit Risiken behaftet sein oder zu Beschwerden führen. Im Einzelnen handelt es sich um die Entnahme von Blut. Bei einer Blutentnahme kann eine Kreislaufreaktion auftreten, die zu Blutdruckveränderung, Schwindelgefühl, Übelkeit und unter Umständen Ohnmacht führen kann. Die Blutentnahmen erfolgen durch Punktion der Vene. Dadurch können gelegentlich lokale Reizungen, wie Schwellungen, Verhärtungen und Entzündungen der Vene und die Bildung von Blutgerinnseln sowie Blutungen in das umliegende Gewebe (Bluterguss), hervorgerufen werden. In sehr seltenen Fällen kann es zu Gefäß- oder Nervenverletzungen kommen.

Bitte teilen Sie den Mitarbeitern der Prüfstelle *alle* Beschwerden, Erkrankungen oder Verletzungen mit, die im Verlauf Ihrer Studienteilnahme auftreten. Falls Sie diese als schwerwiegend empfinden, teilen Sie den Mitarbeitern der Prüfstelle diese bitte umgehend mit, ggf. telefonisch.

I.6. Welche anderen Behandlungsmöglichkeiten gibt es außerhalb der klinischen Prüfung?

Derzeit gibt es keine zugelassenen Behandlungen, die die Barrierefunktion des Darms bei Ihrer Erkrankung wirksam angehen und verbessern kann. Medikamente wie nichtresorbierbare Antibiotika (Rifaximin, Norfloxacin) und Betablocker (Carvedilol, Propranolol) können zwar Auswirkungen auf die Darmmikrobiota und Entzündungsprozesse bei Leberzirrhose haben, werden aber unabhängig von dieser Anwendung bei bestimmten Komplikationen, zum Beispiel nach Peritonitis (Bauchraumentzündung) oder zur Prophylaxe (Vorbeugung) von Blutungen, nach Ermessen Ihres Arztes eingesetzt. Über weitere Einzelheiten kann Sie der Prüfer informieren.

I.7. Wer darf an dieser klinischen Prüfung nicht teilnehmen?

An dieser klinischen Prüfung dürfen Sie nicht teilnehmen, wenn Sie gleichzeitig an anderen klinischen Prüfungen teilnehmen oder in den vergangenen 30 Tagen in einer anderen Prüfung Prüfpräparate erhalten haben.

Schwangere Frauen dürfen an dieser klinischen Prüfung **nicht teilnehmen**.

Zu Beginn der klinischen Prüfung und beim letzten Termin müssen sich deshalb alle Frauen einem Schwangerschaftstest unterziehen. Durch einen Schwangerschaftstest kann jedoch eine Schwangerschaft erst einige Tage nach der Empfängnis verlässlich nachgewiesen werden. Vom Schwangerschaftstest ausgenommen sind Frauen, die nicht mehr schwanger werden können.

Im Falle Ihrer Teilnahme an dieser klinischen Prüfung müssen Sie eine Schwangerschaft zuverlässig verhüten, z.B. durch Enthaltsamkeit, Sterilisation, empfängnisverhütende Implantate (z.B. Spirale oder Verhütungsstäbchen) oder durch eine Kombination von mindestens zwei wirksamen Verhütungsmethoden mit einem Pearl-Index unter 1 %. Das wären z. B. kombinierte hormonelle Verhütungsmittel (Pille, Pflaster oder Vaginalring) mit Barriereverhütung (z.B. Kondome). Diese Methoden sollten Sie während der gesamten Dauer der klinischen Prüfung kontinuierlich anwenden.

Der Grund dafür ist, dass nicht geklärt ist, ob INTESTIFIX 001-Kapseln zu einer Schädigung des Ungeborenen führen können, wenn sie während der Schwangerschaft eingenommen werden.

Sollten Sie während der Studienteilnahme schwanger werden oder den Verdacht haben, dass Sie schwanger geworden sind, müssen Sie umgehend den Prüfer informieren.

Es ist nicht bekannt, ob Bestandteile von INTESTIFIX 001-Kapseln in die Muttermilch gelangen können. Es ist nicht ausgeschlossen, dass Bestandteile der Kapseln in den Körper des Kindes gelangen und das Kind schädigen können. Frauen müssen sich deshalb verpflichten, während der gesamten Dauer der klinischen Prüfung nicht zu stillen.

Männliche Studienteilnehmer sollten ihre Sexualpartner über ihre Teilnahme an der klinischen Prüfung informieren und außerdem bis zum Ende der klinischen Prüfung wirksame Barriereverhütungsmittel (d. h. Kondome) verwenden. Männer müssen außerdem während der gesamten Dauer der klinischen Prüfung auf Samenspenden verzichten.

I.8. Entstehen für mich Kosten durch eine Teilnahme an der klinischen Prüfung? Erhalte ich eine Aufwandsentschädigung?

Durch Ihre Teilnahme an dieser klinischen Prüfung entstehen für Sie ggf. Kosten für die zusätzlichen Anfahrten ans Prüfzentrum.
Sie erhalten die Kosten für die zusätzliche Anfahrt ans Prüfzentrum erstattet. Hierbei bekommen Sie entweder die Kosten für die Hin- und Rückfahrt zum und vom Prüfzentrum mit Ihrem privaten Auto (0,30 €/km) sowie die Parkgebühren oder die Kosten für ein Taxi gegen Vorlage eines Beleges erstattet.

I.9. Bin ich während meiner Teilnahme an der klinischen Prüfung versichert?

Bei der klinischen Prüfung eines Arzneimittels müssen alle Studienteilnehmer gemäß dem Arzneimittelgesetz versichert sein. Der Umfang des Versicherungsschutzes ergibt sich aus den Versicherungsunterlagen, die Sie ausgehändigt bekommen.

Wenn Sie vermuten, dass durch die Teilnahme an der klinischen Prüfung Ihre Gesundheit geschädigt oder bestehende Leiden verstärkt wurden, müssen Sie dies unverzüglich dem Versicherer

Name und Anschrift der Versicherung: [redacted]

Telefon: [redacted]
E-Mail: [redacted]
Versicherungsnummer: [redacted]

direkt anzeigen, gegebenenfalls mit Unterstützung durch Ihren Prüfer, um Ihren Versicherungsschutz nicht zu gefährden. Sofern Ihr Prüfer Sie dabei unterstützt, erhalten Sie eine Kopie der Meldung. Sofern Sie Ihre Anzeige direkt an den Versicherer richten, informieren Sie bitte zusätzlich Ihren Prüfer.

Bei der Aufklärung der Ursache oder des Umfangs eines Schadens müssen Sie mitwirken und alles unternehmen, um weiteren Schaden abzuwenden.

Während der Dauer der klinischen Prüfung dürfen Sie sich einer anderen medizinischen Behandlung – außer in Notfällen – nur nach vorheriger Rücksprache mit dem Prüfer unterziehen. Von einer erfolgten Notfallbehandlung müssen Sie den Prüfer unverzüglich unterrichten.

Sie erhalten ein Exemplar der Versicherungsbestätigung einschließlich der Versicherungsbedingungen. Wir weisen Sie insbesondere auf § 1.4 (zu den Ausschlüssen) und § 1.3 (zum Umfang der Leistungen) hin. Bitte beachten Sie außerdem die Obliegenheiten in § 4 (das sind Pflichten, die Sie im eigenen Interesse beachten müssen, um Ihren Versicherungsschutz zu erhalten).

Wir weisen Sie ferner darauf hin, dass Sie auf dem Weg von und zum Prüfzentrum unter folgender Nummer auf dem direkten Weg von und zum Prüfzentrum bei dem oben genannten Versicherer versichert sind:

Name und Anschrift der Versicherung: [redacted]

[redacted]

Telefon: [redacted]
E-Mail: [redacted]
Versicherungsnummer: [redacted]

I.10. Werden mir neue Erkenntnisse zu der klinischen Prüfung mitgeteilt?

Sie werden während Ihrer Teilnahme über neue Erkenntnisse in Bezug auf diese klinische Prüfung informiert, die für Ihre Bereitschaft zur weiteren Teilnahme wesentlich sein können.

Spätestens ein Jahr nach Beendigung der gesamten klinischen Prüfung werden zusammenfassende Ergebnisse in der europäischen Datenbank (https://euclinicaltrials.eu/search-clinical-trials-reports/) bereitgestellt und können von Ihnen unter der oben angegebenen EU CT-Nr. eingesehen werden. Sie können sich zur Information über die Studienergebnisse zudem gerne an Ihren Prüfer wenden.

I.11. Wer entscheidet, ob ich aus der klinischen Prüfung ausscheide?

Sie können jederzeit, auch ohne Angabe von Gründen, Ihre Studienteilnahme beenden, ohne dass Ihnen dadurch Nachteile entstehen.
Es ist auch möglich, dass der Prüfer oder der Sponsor entscheidet, Ihre Teilnahme an der klinischen Prüfung bzw. zumindest die Einnahme des Prüfpräparats vorzeitig zu beenden, ohne dass Sie auf die Entscheidung Einfluss haben. Die Gründe hierfür können z.B. sein:

- Die Unfähigkeit, das Prüfpräparat zu schlucken;
- eine Verschlechterung Ihrer Lebererkrankung nach bestimmten Kriterien;
- Eine intravenöse Therapie mit Antibiotika (in die Vene verabreicht) oder anderen antimikrobiellen Medikamenten wegen einer Infektion (mit Ausnahme einer einmaligen Antibiotikagabe bei Eingriffen);
- Behandlung mit Medikamenten, welche das Immunsystem beeinflussen (z.B. mit den Wirkstoffen Kortikosteroide, Thiopurine, Calcineurin-Inhibitoren und Mycophenolat).
- Schwangerschaft;
- Ihre weitere Teilnahme an der klinischen Prüfung ist ärztlich nicht mehr vertretbar;
- Die gesamte klinische Prüfung wird abgebrochen.

Wenn Sie die klinische Prüfung vorzeitig beenden, wird Ihnen für Ihre eigene Sicherheit empfohlen, dennoch die Abschlussuntersuchung wahrzunehmen (Termin 5, T5). Die Entnahme von Probenmaterial ist in diesem Fall nicht erforderlich.

Sollte die Behandlung mit der Prüfpräparat bei Ihnen abgebrochen werden, Sie aber nicht das Einverständnis zur Studienteilnahme zurückziehen, ist es für Ihre Sicherheit wichtig, dass Sie trotzdem die Studientermine wahrnehmen.

Der Prüfer wird mit Ihnen besprechen, wie und wo Ihre weitere Behandlung stattfindet.

I.12.An wen wende ich mich bei weiteren Fragen?

Beratungsgespräche an der Prüfstelle
Sie haben stets die Gelegenheit zu weiteren Beratungsgesprächen mit dem auf Seite 1 genannten oder einem anderen Prüfer.

Kontaktstelle
Es existiert außerdem eine Kontaktstelle bei der zuständigen Bundesoberbehörde. Teilnehmer an einer klinischen Prüfung, ihre gesetzlichen Vertreter oder Bevollmächtigten können sich an diese Kontaktstelle wenden:

Bundesinstitut für Arzneimittel und Medizinprodukte
[redacted]

[redacted]

Teil II:
Informationen zum Datenschutz und zu den Biomaterialien

II.1. Was geschieht mit den über mich erhobenen Daten?

a) Allgemeine Informationen

Während der klinischen Prüfung werden medizinische Befunde (z.B. Gesundheitsdaten) und persönliche Informationen (z.B. Alter, Geschlecht, ethnische Herkunft) von Ihnen erhoben und in der Prüfstelle in Ihrer persönlichen Akte niedergeschrieben und/oder elektronisch gespeichert. Die Aufzeichnung der im Rahmen dieser klinischen Prüfung erhobenen Daten erfolgt zunächst in Originalunterlagen bzw. Ihrer Krankenakte, in die Ihr Prüfer auch bisher alle Befunde eingetragen hat. Die für die klinische Prüfung wichtigen Daten werden zusätzlich, zur Dokumentation in der Krankenakte, auch in pseudonymisierter (verschlüsselter) Form gespeichert.

Pseudonymisiert bedeutet, dass keine Angaben, mit denen Sie direkt identifiziert werden können (z.B. Namen, Kontaktinformationen, Geburtsdatum) verwendet werden, sondern nur ein Nummern- und/oder Buchstabencode.

Ihr Prüfer erstellt eine Pseudonymisierungsliste, die gegen unbefugten Zugriff geschützt am Prüfzentrum verbleibt. Das ist notwendig, damit Ihnen diese personenbeziehbaren Daten, falls erforderlich, wieder zugeordnet werden können. Eine solche Entschlüsselung erfolgt nur, unter den vom Gesetz beschriebenen Voraussetzungen, insbesondere in folgenden Fällen: Für die Rückverfolgbarkeit und Vernichtung der Daten und Restmaterial der Proben bei Widerruf Ihrer Einwilligung. Der Schlüssel zu diesem Code verbleibt stets beim Prüfzentrum.

Die pseudonymisierten Daten werden insbesondere an den Sponsor dieser klinischen Prüfung zur Auswertung weitergegeben und dort gespeichert (siehe II. 1. Buchst. e) Weitergabe/Empfänger).

Es ist für die Durchführung der klinischen Prüfung ebenfalls erforderlich, dass personenbezogene Daten aus vorangegangenen Untersuchungen an Ihnen oder aus Untersuchungen von Ärzten außerhalb der Prüfstelle zu Ihrer persönlichen Akte hinzugefügt werden. Daher kann Ihr Prüfer Ihren Hausarzt und/oder mitbehandelnde Ärzte kontaktieren, um diese Informationen zu ergänzen. Mit Ihrer Einwilligung zur Teilnahme an der klinischen Prüfung entbinden Sie Ihren Hausarzt bzw. die behandelnden Ärzte von ihrer Schweigepflicht.

b) Rechtsgrundlage

Rechtsgrundlage für die Datenverarbeitung ist Ihre informierte Einwilligung gemäß Art. 9 Abs. 2 Buchst. a und Art. 6 Abs. 1 Buchst. a der EU-Datenschutzgrundverordnung (DSGVO) sowie gemäß § 40b Abs. 6 Arzneimittelgesetz, und Art. 9 Abs. 2 Buchstabe j DSGVO.

Die Bereitstellung Ihrer personenbezogenen Daten ist freiwillig. Ohne Ihre ausdrückliche Einwilligung in die Verarbeitung Ihrer Daten können Sie allerdings nicht an dieser klinischen Prüfung teilnehmen.

c) Verantwortlichkeit

Verantwortlich im Sinne des Datenschutzrechts ist der Sponsor ebenso wie die Prüfstelle und Ihr Prüfer. Die Prüfstelle bleibt davon unabhängig für Ihre Behandlungsdaten verantwortlich (unkodierte Patientendaten).

Die klinische Prüfung wird veranlasst und organisiert durch das Universitätsklinikum Aachen für die Medizinische Fakultät der RWTH Aachen, den gesetzlichen Sponsor dieser klinischen Prüfung. Der Sponsor ist gemäß Art. 26 DSGVO gemeinsam mit der Prüfstelle bzw. Ihrem Prüfer für die Datenverarbeitung im Sinne der Datenschutzgrundverordnung (DSGVO) verantwortlich. Dabei trägt der Sponsor in der Hauptsache die Verantwortung für die pseudonymisierten Daten. Das Prüfzentrum ist für Ihre unkodierten Patientendaten verantwortlich.

d) Zweck(e)

Mit Hilfe der erhobenen Daten soll die Sicherheit von INTESTIFIX 001-Kapseln bei Menschen mit Leberzirrhose mit Aszites klinisch untersucht werden.
Zudem werden die erhobenen Daten auch bei den notwendigen Zulassungsverfahren für das zu untersuchende Arzneimittel verwendet.
Zusätzlich sollen die von Ihnen entnommenen Proben oder Ihre erhobenen Daten für weitere Zwecke innerhalb dieser Studie ausgewertet werden. Dabei werden insbesondere die explorativen Endpunkte (weiterführende Forschung innerhalb dieser klinischen Prüfung), einschließlich der Darmmikrobiota, von Arbeitsgruppen des von der Deutschen Forschungsgemeinschaft (DFG) geförderten Sonderforschungsbereich 1382 „Darm-Leber-Achse" weitergehend analysiert. Außerdem werden gegebenenfalls Restmaterialien der Proben aufbewahrt und für weitere Fragestellungen außerhalb dieser klinischen Prüfung verwendet, sofern Sie dem zustimmen.

e) Weitergabe/Empfänger

Die für die klinische Prüfung wichtigen Daten und Biomaterial werden zusätzlich in pseudonymisierter Form verarbeitet und gegebenenfalls weitergegeben.
Wenn es erforderlich ist, die von Ihnen erhobenen Daten weiterzugeben, dann erfolgt dies in pseudonymisierter Form und ausschließlich an folgende Stellen:

1. den Sponsor Universitätsklinikum Aachen, AöR und von diesem beauftragte Stellen zum Zweck der Durchführung und wissenschaftlichen Auswertung, Bewertung oder Veröffentlichung.
2. im Falle unerwünschter Ereignisse:
    - an den Sponsor,
    - die jeweilige zuständige Ethikkommission,
    - andere Prüfer,
    - sowie von diesem ggf. an die zuständigen Behörden der Mitgliedstaaten der Europäischen Union oder des Abkommens über den Europäischen Wirtschaftsraum, in deren Hoheitsgebiet die o.g. klinische Prüfung durchgeführt wird
    - oder an die für die Überwachung der Arzneimittelsicherheit eingerichtete Europäische Datenbank (EudraVigilance), auf die die zuständigen Überwachungsbehörden in der gesamten Europäischen Union und dem Europäischen Wirtschaftsraum Zugriff haben.
    - an den Hersteller des Prüfpräparates
3. im Fall eines Antrags auf Zulassung als Arzneimittel an den Antragsteller und die für die Zulassung zuständige Behörde,

4. für die weitere Auswertung der weiterführenden Endpunkte (explorative Endpunkte) innerhalb dieser klinischen Prüfung werden Daten und Proben an die Forschungslabore des SFB 1382 „Darm-Leber-Achse“ innerhalb der medizinischen Fakultät des Universitätsklinikums Aachen weitergegeben.

Die von Ihnen im Rahmen der oben genannten klinischen Prüfung erhobenen und gespeicherten Daten (auch Ihre Originaldaten in der Krankenakte) können, soweit erforderlich und gesetzlich erlaubt, durch die zuständige Überwachungsbehörde im Rahmen von Inspektionen oder Beauftragte des Sponsors (sogenannte Auditoren oder Monitore) zur Überprüfung der ordnungsgemäßen Durchführung der klinischen Prüfung eingesehen werden. Diese sind zur [redacted] verpflichtet, eine Weitergabe der erhobenen Daten erfolgt in diesem Zusammenhang nicht.

Im Rahmen dieser klinischen Prüfung erfolgt eine Weitergabe Ihrer pseudonymisierten Daten zum Zweck der Datenauswertung, Zulassung und Überwachung nur innerhalb der Europäischen Union und des Europäischen Wirtschaftsraumes.

f) Ihre Rechte

Sie haben grundsätzlich folgende Rechte bezüglich ihrer personenbezogenen Daten, sofern dies nicht aufgrund einer zwischenzeitlich vorgenommenen Löschung der identifizierenden Merkmale zur Entschlüsselung technisch oder anderweitig gesetzlich unmöglich ist:

Recht auf Widerruf ihrer Einwilligung und Löschung Ihrer personenbezogenen Daten:

So wie die Einwilligung zur Teilnahme an der klinischen Prüfung können Sie auch Ihre Einwilligung zur Verarbeitung der erhobenen Daten jederzeit widerrufen. Gemäß § 40b Abs. 6 Nr. 2 AMG dürfen im Falle eines Widerrufs Ihre gespeicherten Daten jedoch weiterverwendet werden, soweit dies erforderlich ist, um

1. die Wirkungen/Sicherheit des zu prüfenden Arzneimittels festzustellen,
2. sicherzustellen, dass Ihre schutzwürdigen Interessen nicht beeinträchtigt werden,
3. der Pflicht zur Vorlage vollständiger Zulassungsunterlagen zu genügen.

Im Falle eines Widerrufs Ihrer Einwilligung werden die verantwortlichen Stellen unverzüglich prüfen, inwieweit die gespeicherten Daten noch erforderlich sind. Nicht mehr benötige Daten werden unverzüglich gelöscht, sofern nicht gesetzliche und/oder behördliche Dokumentations- und Meldepflichten entgegenstehen. Die bis zum Widerruf erfolgte Datenverarbeitung bleibt jedoch rechtmäßig.

Sie haben weiterhin folgende Rechte:

- Recht auf Auskunft (inkl. unentgeltlicher Überlassung einer Kopie) über Ihre personenbezogenen Daten, die im Rahmen der klinischen Prüfung erhoben, verarbeitet oder ggf. an Dritte übermittelt werden.
- Recht auf Datenübertragung der zu Ihrer Person erhobenen Daten an eine bestimmte Stelle.
- Recht auf Berichtigung unrichtiger personenbezogener Daten.
- Recht auf Einschränkung der Verarbeitung.
- Recht auf Widerspruch gegen die Nutzung der Daten.

Mögliche Einschränkungen Ihrer Rechte

Um jedoch die wissenschaftliche Integrität der klinischen Prüfung zu schützen, müssen die Behandlung, die Sie in der klinischen Prüfung erhalten, und die Ergebnisse der Behandlung möglicherweise vor Ort verbleiben, bis die Studiendaten analysiert wurden. Sie können daher möglicherweise Ihre Rechte in Bezug auf Ihre personenbezogenen Daten erst ausüben, wenn die gesetzlichen Verpflichtungen des Sponsors erfüllt sind (d.h. bis die Studiendaten analysiert wurden).

Wahrnehmung Ihrer Rechte

Wenn Sie Fragen oder Bedenken bezüglich des Datenschutzes und der Verwendung Ihrer persönlichen Gesundheitsdaten haben, wenden Sie sich an Ihren Prüfer oder an die folgenden Datenschutzbeauftragten:

Datenschutzbeauftragter des Prüfzentrums:
[redacted]

Datenschutzbeauftragter des Sponsors:
Siehe oben

Sie haben grundsätzlich auch das Recht, jederzeit den Sponsor selbst zu kontaktieren. Bitte wenden Sie sich jedoch im Regelfall an den Prüfer bzw. den Datenschutzbeauftragten Ihres Prüfzentrums, da aufgrund der Pseudonymisierung nur hier Ihre Identität bekannt ist und damit sinnvollerweise weitere Schritte unternommen werden können, bzw. eine unbeabsichtigte Identifikation Ihrer Person durch den Sponsor vermieden werden kann.
Wir nehmen Ihre Anliegen und Rechte sehr ernst. Wenn Sie jedoch der Meinung sind, dass im Rahmen dieser klinischen Prüfung eine Verletzung Ihres Datenschutzes vorliegt, haben Sie das Recht sich an einer der zuständigen Datenschutzaufsichtsbehörden zu wenden:

Datenschutzaufsichtsbehörde des Bundeslandes, in dem Ihr Prüfzentrum liegt:
[redacted]

Für den Sponsor bzw. dessen Vertreter innerhalb der EU zuständige Datenschutzaufsichtsbehörde:
Siehe oben

Eine Liste aller in Deutschland und der Europäischen Union zuständigen Datenschutz-aufsichtsbehörden finden Sie hier:
https://www.bfdi.bund.de/DE/Infothek/Anschriften_Links/anschriften_links-node.html

g) Dauer der Speicherung der Daten:

Die erhobenen Daten werden von der Prüfstelle und dem Sponsor über den gesetzlich geforderten Zeitraum sicher aufbewahrt und daher für die Dauer von 25 Jahren nach Beendigung oder Abbruch der klinischen Prüfung gespeichert. Danach werden Ihre personenbezogenen Daten gelöscht.

h) Veröffentlichung
Wissenschaftliche Veröffentlichungen von Ergebnissen erfolgen in einer anonymisierten Form, die keine direkten Rückschlüsse auf Ihre Person zulässt.

II.2. Was geschieht mit meinen Biomaterialien?

Während dieser klinischen Prüfung wird Ihnen Biomaterial (Blut, Stuhl, ggf. Bauchwasser) entnommen. Die Proben werden ebenso wie die Daten in pseudonymisierter Form aufbewahrt (siehe zur Pseudonymisierung oben II. 1. Buchst. a).
Die Pseudonymisierung Ihres Biomaterials bietet allerdings nicht zwangsläufig den gleichen Schutz wie die Pseudonymisierung der erhobenen Daten. Ihr Biomaterial beinhaltet immer Informationen zu Ihrer Erbsubstanz, die eine Identifizierung ermöglichen könnten. Zudem sind in Ihrem Biomaterial sensible genetische Informationen gespeichert, die etwa Aussagen zu Verwandtschaftsbeziehungen oder auch Erbkrankheiten zulassen. Ein Identifizierungsrisiko lässt sich nicht völlig ausschließen und steigt, je mehr Daten miteinander verknüpft werden können.

Da somit aus Ihren Biomaterialien Informationen gewonnen werden können, gelten die oben unter II. 1. genannten datenschutzrechtlichen Aspekte entsprechend auch für Ihre Biomaterialien.

a) Verwendung Ihrer Biomaterialien
Ihre Biomaterialien werden für diese klinische Prüfung und möglicherweise auch für weitere Fragestellungen im Zusammenhang mit INTESTIFIX 001-Kapseln verwendet. (siehe dazu auch Punkt II. 1. d) Zwecke). Mit solchen weitergehenden Untersuchungen sollen Fragen geklärt werden, die sich noch im weiteren Verfahren der Arzneimittelzulassung stellen können. Insbesondere werden Ihre Proben für die Analyse der explorativen Endpunkte (weiterführende Forschung innerhalb dieser klinischen Prüfung), einschließlich der Darmmikrobiota, vom SFB (Sonderforschungsbereich) 1382 „Darm-Leber-Achse" verwendet. Außerdem werden gegebenenfalls Restmaterialien der Proben aufbewahrt und für weitere Fragestellungen außerhalb dieser klinischen Prüfung verwendet. Hierfür erhalten Sie eine weitere Patienteninformation und Einwilligungserklärung. Die Zustimmung zur weiteren Verwendung außerhalb dieser Studie ist unabhängig von der Verwendung der Biomaterialien für diese Studie.

Dessen ungeachtet bleiben Ihre Rechte, die Ihre Persönlichkeit im Zusammenhang mit dem Körpermaterial schützen, davon unberührt.

b) Lagerung
Die Aufbewahrung der Biomaterialien erfolgt unter Verantwortung von Univ.-Prof. Dr. med. Tony Bruns. Falls Sie dem zustimmen (siehe separate Patienteninformation und Einwilligungserklärung), werden Restmaterialien Ihrer Biomaterialien nach Abschluss der studienspezifischen Analysen - für weitere Zwecke (außerhalb dieser klinischen Prüfung) aufbewahrt, verwendet und ausgewertet. Sollten Sie dem nicht zustimmen, werden sie nach Durchführung aller studienbedingten Analysen vernichtet, soweit

keine gesetzliche Aufbewahrungspflicht besteht. Eventuelle Restmaterialien der Proben werden in den Laboren des Sonderforschungsbereichs „Darm-Leber-Achse" innerhalb der Medizinischen Fakultät des Universitätsklinikums Aachen aufbewahrt.

Ihre Biomaterialien werden unter Verantwortung von Univ.-Prof. Dr. med. Tony Bruns ausschließlich innerhalb der Europäischen Union gelagert.

c) Weitergabe/Empfänger

Ihre Biomaterialien werden an folgende Empfänger weitergegeben: Forschungslabor des Lehr- und Forschungsgebiets Hepatologie, Universitätsklinikum Aachen, Anstalt des öffentlichen Rechts (AöR), Pauwelsstraße 30, 52074 Aachen. Außerdem kann eine Weitergabe an die Forschungslabore des SFB 1382 „Darm-Leber-Achse" innerhalb der medizinischen Fakultät des Universitätsklinikums Aachen erfolgen. Es gelten entsprechend die Ausführungen unter II. 1. Buchst. e).

d) Umgang mit dem Biomaterial bei Widerruf/vorzeitiger Beendigung der Teilnahme

Wenn Sie Ihre Teilnahme an der klinischen Prüfung vorzeitig beenden möchten, werden Ihre Proben zum Erreichen der Studienziele weitergehend analysiert. Restmaterialien der Proben werden im Anschluss an die Beendigung aller studienrelevanter Untersuchungen umgehend vernichtet. Der Sponsor ist für die Vernichtung der Proben verantwortlich.

Einwilligungserklärung

Titel der Klinischen Prüfung:
Verkapselter fäkaler Mikrobiota-Transfer zur Behandlung der Immunaktivierung bei Patienten mit Zirrhose und Aszites (TransImmune) - Eine randomisierte, doppelblinde, placebokontrollierte klinische Pilotstudie der Phase IIa

______________________________ ______________________________
Name Patient in Druckbuchstaben **Geburtsdatum Patient**

Ich bin in einem persönlichen Gespräch durch den Prüfer

____
Name Hauptprüfer/Prüfer in Druckbuchstaben

ausführlich und verständlich über Wesen, Bedeutung, Risiken und Tragweite der Studienteilnahme aufgeklärt worden. Ich habe darüber hinaus den Text der Patienteninformation mit seinen beiden Teilen (Teil I: Informationen zu gesundheitlichen Aspekten; Teil II: Informationen zur Verwendung der Daten und Biomaterialien) gelesen und verstanden. Ich hatte die Gelegenheit, mit dem Prüfer über die Durchführung der klinischen Prüfung zu sprechen. Alle meine Fragen wurden zufriedenstellend beantwortet.

Ich hatte ausreichend Zeit, mich zu entscheiden.

Mir ist bekannt, dass ich jederzeit und ohne Angabe von Gründen meine Einwilligung zur Teilnahme an der klinischen Prüfung zurückziehen kann (mündlich oder schriftlich), ohne dass mir daraus Nachteile für meine medizinische Behandlung entstehen.

Möglichkeit zur Dokumentation zusätzlicher Fragen seitens des Patienten oder sonstiger Aspekte des Aufklärungsgesprächs:

____

____

____

____

Datenschutzrechtliche Einwilligung

I. Mir ist bekannt, dass bei dieser klinischen Prüfung **personenbezogene Daten**, insbesondere medizinische Befunde, über mich erhoben, gespeichert und ausgewertet werden sollen. Die Verwendung meiner personenbezogenen Daten setzt vor der Teilnahme an der klinischen Prüfung folgende freiwillig abgegebene Einwilligungserklärung voraus; ohne die nachfolgende Einwilligung kann ich nicht an der klinischen Prüfung teilnehmen.

1. Ich willige ein, dass im Rahmen dieser klinischen Prüfung personenbezogene Daten, insbesondere Angaben über meine Gesundheit und meine ethnische Herkunft, über mich – wie zuvor beschrieben und für den genannten Zweck – erhoben und in Papierform sowie auf elektronischen Datenträgern gemäß den Angaben in der Informationsschrift Teil II 1. aufgezeichnet, verwendet und weitergegeben (verarbeitet) werden dürfen. Die dort genannten gesetzlichen Einschränkungen meiner Rechte sind mir bewusst.

2. Soweit erforderlich, dürfen die erhobenen Daten pseudonymisiert (verschlüsselt) weitergegeben werden:
a) an den Sponsor oder eine von diesem beauftragte Stelle zum Zwecke der wissenschaftlichen Auswertung, Bewertung und Veröffentlichung;
b) im Falle eines Antrags auf Zulassung: an den Antragsteller und die für die Zulassung zuständige Behörde (z. B. das Bundesinstitut für Arzneimittel und Medizinprodukte [BfArM] oder andere zuständige nationale Behörden);
c) im Falle unerwünschter Ereignisse: an den Sponsor, andere Prüfer, an die jeweils zuständige Ethikkommission und die zuständigen Behörden der Mitgliedstaaten der Europäischen Union oder des Abkommens über den Europäischen Wirtschaftsraum, in deren Hoheitsgebiet die vorgenannte klinische Prüfung durchgeführt wird, und an die europäische Datenbank (EudraVigilance), auf die auch die zuständigen Behörden der anderen Mitgliedstaaten der Europäischen Union und des Abkommens über den Europäischen Wirtschaftsraum Zugriff haben.
d) Im Fall von unerwünschten Ereignissen an den Hersteller des Prüfpräparates

3. Ich bin darüber aufgeklärt worden, dass ich jederzeit meine Teilnahme an der klinischen Prüfung beenden kann. Die Einwilligung in die Erhebung und Verarbeitung meiner personenbezogenen Daten, insbesondere der Angaben über meine Gesundheit, ist jedoch unwiderruflich. Ich weiß, dass im Falle eines Widerrufs der Einwilligung in die Teilnahme an der klinischen Prüfung, die bis zu diesem Zeitpunkt gespeicherten Daten ohne Nennung meines Namens weiterhin verwendet werden dürfen, soweit dies erforderlich ist, um
a) Wirkungen/Sicherheit des zu prüfenden Arzneimittels festzustellen,
b) sicherzustellen, dass meine schutzwürdigen Interessen nicht beeinträchtigt werden,
c) der Pflicht zur Vorlage vollständiger Zulassungsunterlagen zu genügen.

4. Ich bin damit einverstanden, dass mein Hausarzt ______________________ (Name des Hausarztes) über meine Teilnahme an dieser klinischen Prüfung informiert wird.

5. Ich willige ein, dass Gesundheitsdaten von mitbehandelnden Ärzten erhoben werden, soweit dies für die ordnungsgemäße Durchführung und Überwachung der klinischen Prüfung notwendig ist. Insoweit entbinde ich diese Ärzte von der Schweigepflicht.

II.

1. Ich willige ein, dass meine **Biomaterialien** - für den genannten Zweck - gemäß den Angaben der Informationsschrift Teil II 2. beschrieben aufbewahrt, verwendet und weitergegeben werden.

Das Eigentum an den Biomaterialien übertrage ich an den Sponsor der Studie

Einwilligung in die Studienteilnahme

Ich willige freiwillig ein, an der oben genannten klinischen Prüfung teilzunehmen. Zugleich willige ich in die Verarbeitung meiner personenbezogenen Daten und Biomaterialien wie oben beschrieben und von mir angegeben ein.

**Name Patient** in Druckbuchstaben	**Unterschrift Patient**
**Ort, Datum** der Unterschrift des **Patienten**	**Uhrzeit** der Unterschrift des **Patienten**

Ich habe das Aufklärungsgespräch geführt und die Einwilligung des Patienten eingeholt. Ich werde dem Patienten nach meiner Unterzeichnung ein Exemplar der Patienteninformation und der unterschriebenen Einwilligungserklärung zur Studienteilnahme sowie eine Kopie der Versicherungsbestätigung und der Versicherungsbedingungen aushändigen.

**Name aufklärender Hauptprüfer/Prüfer** in Druckbuchstaben	**Unterschrift aufklärender Hauptprüfer/Prüfer**
**Ort, Datum** der Unterschrift des **aufklärenden Hauptprüfers/Prüfers**	**Uhrzeit** der Unterschrift des **Hauptprüfers/Prüfers**
