# Supplementary material for: Encapsulated faecal microbiota transfer to target immune activation in patients with cirrhosis and ascites (TransImmune): protocol for a randomised, double-blind, Phase IIa, placebo-controlled trial
Source: BMJ Open. 2026 Aug 12;16(8):e119299. doi: 10.1136/bmjopen-2026-119299 (PMC13475211; doi:10.1136/bmjopen-2026-119299)
Supplement: Supplementary file 2 [file bmjopen-2026-119299.reviewer_comments.pdf]

PEER REVIEW HISTORY

BMJ Open publishes all reviews undertaken for accepted manuscripts. Reviewers are asked to complete a checklist review form and are provided with free text boxes to elaborate on their assessment. These free text comments are reproduced below.

ARTICLE DETAILS

Title (Provisional)

Encapsulated Faecal Microbiota Transfer to Target Immune Activation in Patients with Cirrhosis and Ascites (TransImmune): Protocol for a randomised, double-blind, phase IIa, placebo-controlled trial

Authors

Große, Karsten; Haedge, Frederic; Fera, Carmen; Hecker, Jana; Wienstroer, Jan; Tsakmaklis, Anastasia; Fichtner, Andreas; Treichel, Nicole S; Clavel, Thomas; Wirtz, Theresa Hildegard; Pabst, Oliver; Schuckelt, Rainer; Vehreschild, Maria JGT; Bruns, Tony

---

VERSION 1 - REVIEW

**Reviewer**	**1**
**Name**	**Thomsen, Karen Louise**
**Affiliation**	**Aarhus University Hospital, Department of Hepatology and Gastroenterology**
**Date**	**08-May-2026**
**COI**	**None**

The TransImmune trial's main aim is to evaluate whether treatment with encapsulated FMT is safe in patients with decompensated cirrhosis and ascites. The planned study is highly interesting; a few aspects of the protocol could be elaborated on and clarified:

In the FMT society, the agreed-on terminology for FMT is that it is an abbreviation for Faecal Microbiota Transplantation. Faecal Microbiota Transfer has previously been used by some centres, any specific reason why Transfer is used here? – should be mentioned, alternatively changed to Transplantation.

In the Introduction in the section on Rational for the TransImmune Trial, percentages of patients experiencing AE's and SAE's after FMT in patients with cirrhosis is mentioned and suggested to be higher than in non-cirrhotics, please add numbers for AE's and SAE's in non-cirrhotic c. diff. patients after FMT for easy interpretation.

Objectives:

Primary objective: 'The occurrence of serious adverse events (SAE) up to the end of the study (EOS)', please specify if this means at EOS (V5) or also at V3 and V4.

Secondary objectives: is peritoneal inflammation not missing?

The planned analyses for investigation 'immune activation' e.g. systemic inflammation, gut inflammation/intestinal barrier integrity and peritoneal inflammation seem limited, but probably due to the small cohort of patients. However, it could be considered to include more analyses investigating immune activation, as the trial and collected samples are a golden opportunity to investigate these matters further.

Inclusion criteria:

Please elaborate on part of the main inclusion criterium of having ascites. Having ascites requiring therapeutic paracentesis and needing a diagnostic ascitic tap can be two very different patients e.g. being acutely decompensated or having small amounts of ascites with suspected SBP.

Patients requiring a therapeutic paracentesis or a diagnostic ascitic tap within 30 days prior to inclusion can be included. Please clarify if it is not mandatory to rule out SBP with a diagnostic puncture just before inclusion?

Recruitment and randomisation:

Randomisation will be performed using a permuted block design. Please specify how many patients in each block?

FMT preparation:

Please specify if one FMT preparation (IMP) is using faeces from only one donor or donations are pooled from different donors? How many different donors are planned to be used in the study for the 12 FMT-treated patients? How does using this FMT preparation minimize batch-to-batch variability as mentioned in the Implications section?

Quality control is performed to ensure sufficient diversity defined as a Shannon Diversity Index >2 and Richness >30 genera. Please add or explain why these numbers should be sufficient? Sufficient to achieve what? Importantly, what data are these numbers based on?

Intervention:

'Three daily doses of eight capsules', please specify that the eight capsules are given at the same time/once daily on three consecutive days.

The dose was selected 'to optimize the probability of microbial engraftment and barrier restoration', what data is this dose based on?

'To ensure product viability, the IMP is removed from ultra-low temperature storage 10 to 30 minutes prior to administration'. Please clarify what is meant by viability?

The first three randomised patients will be treated sequentially. Can you be sure that there will be FMT treated patients in the first three patients included? How big are the blocks in the block design?

In the sentence 'However, IMP administration must be permanently discontinued if .. etc.': suggest to add 'during the treatment phase' in the end of the sentence, as it can be misunderstood as if the treatment were going on for longer than just the three consecutive days in the beginning.

Follow-up:

Peritoneal inflammation is mentioned as an exploratory objective. Please clarify if collection of ascites is only performed if patients need a paracentesis at follow-up and is not collected at

baseline for comparison pre-FMT? In the legend for Figure 1 (Patient flow chart) it is written that Ascites fluid will be collected at scheduled visits.

Audits and inspections:

'Audits will be conducted according to the study-specific audit plan. This may include on-site visits by a member of the sponsor's quality assurance unit or an authorized independent auditor'. Whether the audits are conducted by a person working for the sponsor or an independent authority ought to be decided before start of trial.

Safety:

It is mentioned that an independently DSMB will periodically review safety data. It is recommended to specify before start of trial how often / after how many patients included (or according to other criteria) this happens.

**Reviewer**	**2**
**Name**	**O'Brien, Alastair**
**Affiliation**	**UCL, Division of Medicine**
**Date**	**05-Jun-2026**
**COI**	**None**

I enjoyed reading about this exciting study and look forward to the results in due course.

I have a few minor comments for the team to address.

Good luck with the study

Strengths and limitations - line 64: I would suggest adding very to statistical power is limited.

Line 67-9: I agree with their pragmatic "real-world" approach but given 12 per group - even with randomisation there is a high risk these medications will not be balanced between groups. Of course, this does not matter for primary endpoint of safety.

Line 81: I would suggest describing this as a key mechanism as central to progression is the driver for disease - most commonly alcohol and then MASLD in decompensated cirrhosis.

Line 198 - is alcohol consumption permitted

Line 278: It states patients will be watched swallowing the capsules - are the patients hospitalised for these 3 days or is this via mobile phone footage?

Do the team plan an ascitic tap within 48 hrs of first dosing to exclude SBP?

Line 283 - what time gap between treating the first 3 patients is planned to observe AEs - ?1 week

---

VERSION 1 - AUTHOR RESPONSE

Reviewer 1 comments:

7. In the FMT society, the agreed-on terminology for FMT is that it is an abbreviation for Faecal Microbiota Transplantation. Faecal Microbiota Transfer has previously been used by some centres, any specific reason why Transfer is used here? – should be mentioned, alternatively changed to Transplantation.

   Response: We thank the reviewer for this thoughtful observation regarding terminology. We are aware that "Faecal Microbiota Transplantation" is the term most commonly endorsed in the international FMT community. Our use of "Faecal Microbiota Transfer" is a deliberate choice that reflects the established terminology in the German-speaking medical community, where this study is conducted. In Germany, faecal microbiota transfer is the conventional designation used in the national medical literature (Stallmach et al., Fecal Microbiota Transfer - Reliable indications, donor screening and modes of application. Dtsch Arztebl Int 2020; 117: 31-8. DOI: 10.3238/arztebl.2020.0031). The preference for "Transfer" over "Transplantation" in this context also reflects the convention that the term "transplantation" is better reserved for the grafting of solid organs and tissues. We therefore suggest to keep this terminology.

8. In the Introduction in the section on Rational for the TransImmune Trial, percentages of patients experiencing AE's and SAE's after FMT in patients with cirrhosis is mentioned and suggested to be higher than in non-cirrhotics, please add numbers for AE's and SAE's in non-cirrhotic c. diff. patients after FMT for easy interpretation.

   Response: We thank the reviewer for this suggestion. The section reads as follows: "Establishing the safety profile in this specific cohort is paramount. Currently, the majority of FMT safety data is derived from patients without liver disease (e.g., CDI), where adverse events (AEs) occur in approximately 19% of procedures (mostly mild diarrhoea or abdominal pain), with serious adverse events (SAEs) remaining rare at 1.4%[29].

   Regarding immunocompromised patients, current literature generally supports FMT application due to an acceptable risk profile30, yet severe outcomes, including treatment-related infections, colectomies, and death, have been observed in isolated cases among severely immunosuppressed patients[31]. In patients with cirrhosis, the safety landscape is more nuanced. In a multicentre cohort of patients with cirrhosis undergoing FMT for CDI, FMT-related AEs occurred in 30.2% (most self-limited abdominal cramping or diarrhoea) and FMT-related SAEs in

9. Primary objective: 'The occurrence of serious adverse events (SAE) up to the end of the study (EOS)', please specify if this means at EOS (V5) or also at V3 and V4.

   Response: We thank the reviewer for this helpful request for clarification. SAEs are continuously assessed and recorded throughout the entire study period, at every study

visit (including V3 and V4), and not only at the EOS. The phrase "up to the end of the study (EOS)" refers to the duration of the observation period. That is, SAEs are captured from inclusion until the EOS visit (V5, day 90+14), which defines the end of safety follow-up.

To avoid any ambiguity, we have revised the description of the primary endpoint to read: “The occurrence of serious adverse events (SAEs) recorded at any time during the study, from inclusion until the end of study (EOS) visit”

10. Secondary objectives: is peritoneal inflammation not missing?
    Response: We thank the reviewer for this careful observation. The assessment of peritoneal inflammation is intentionally not listed among the secondary endpoints but is part of the exploratory objectives of the trial. The revised manuscript reads: “The exploratory objectives of this trial aim to evaluate the effectiveness of strain engraftment from the FMT products by shotgun metagenomic sequencing, the longevity of the graft, and how these factors affect the intestinal barrier function, as well as systemic and peritoneal inflammation. Exploratory analyses include but are not limited to serum and ascitic fluid markers of immune cell activation, systemic inflammation, acute-phase response, and immune activation; the composition of the faecal microbiome, virome, and mycobiome by shotgun metagenomic sequencing; circulating and faecal markers of intestinal permeability, colonic inflammation, and bacterial translocation; and the frequency, differentiation, cytokine repertoire, and cytotoxicity of immune cell subsets in blood and, where clinically indicated paracentesis is performed, in ascitic fluid—each assessed longitudinally from baseline (day 0) through day 7 (±2), day 28 (±3), and end of study (day 90 ± 14).”

11. The planned analyses for investigation ‘immune activation’ e.g. systemic inflammation, gut inflammation/intestinal barrier integrity and peritoneal inflammation seem limited, but probably due to the small cohort of patients. However, it could be considered to include more analyses investigating immune activation, as the trial and collected samples are a golden opportunity to investigate these matters further.
    Response: We thank the reviewer for this encouraging and insightful comment, and we fully share the view that this trial and the associated biospecimens represent a valuable opportunity to characterise immune activation in this population in greater depth. The analyses described in the manuscript represent the predefined primary and secondary endpoints of the trial. To investigate immune activation more comprehensively, we have prospectively collected and biobanked a broad panel of biomaterials across multiple compartments (blood, serum, plasma, stool, and ascitic fluid) at each study visit for exploratory study endpoints. These samples will allow us to pursue a range of additional, more mechanistic analyses beyond the predefined endpoints. We deliberately refrained

from providing an exhaustive list of all planned and potential analytical methods in the manuscript, in order to keep the protocol focused and to avoid overloading the text.

12. requiring therapeutic paracentesis and needing a diagnostic ascitic tap can be two very different patients e.g. being acutely decompensated or having small amounts of ascites with suspected SBP.
    Patients requiring a therapeutic paracentesis or a diagnostic ascitic tap within 30 days prior to inclusion can be included. Please clarify if it is not mandatory to rule out SBP with a diagnostic puncture just before inclusion?
    Response: The 30-day window defines eligibility based on the presence of clinically relevant ascites and is intended to capture both presentations described by the reviewer. Importantly, ascitic paracentesis is **not** a study-defined procedure. A dedicated, study-mandated diagnostic tap immediately prior to inclusion is therefore **not required**.
    Ruling out SBP is governed by standard of care: paracentesis is performed on unplanned hospitalisation, new or worsening decompensation, new organ dysfunction, or clinical suspicion of infection. In accordance with German and institutional guidelines, patients routinely undergo an ascitic tap on admission to exclude SBP; a positive finding precludes enrolment. Active infection, including SBP, is thus reliably excluded through routine clinical assessment rather than through an additional study-specific puncture.

13. Randomisation will be performed using a permuted block design. Please specify how many patients in each block?
    Response: We thank the reviewer for this question. The specific block size(s) are intentionally not disclosed. Withholding the block size is a deliberate safeguard against selection bias, as knowledge of a fixed block size would allow allocation of the final positions in each block to be predicted, thereby compromising allocation concealment. We can confirm that a permuted block design with an appropriate block size is used, and full details are documented in the sealed randomisation plan held by the statistician.

14. Please specify if one FMT preparation (IMP) is using faeces from only one donor or donations are pooled from different donors? How many different donors are planned to be used in the study for the 12 FMT-treated patients? How does using this FMT preparation minimize batch-to-batch variability as mentioned in the Implications section?
    Response: We thank the reviewer for this request for clarification. INTESTIFIX 001 is not manufactured from pooled faecal material from different donors; it is manufactured from material sourced from the Cologne Microbiota Bank (CMB) donor pool. Importantly, each released "treatment" is derived from material from one donation of one qualified donor. Therefore, individual study participants will receive capsules derived from different donors across the trial. Based on the current manufacturing plan, approximately three qualified

donors are expected to cover the 12 FMT-treated patients. The previous wording could be misinterpreted regarding what "batch-to-batch variability minimization" means. The statement that INTESTIFIX 001 minimizes batch-to-batch variability refers specifically to variability introduced by manufacturing, release testing, storage, and shipment procedures and not to the elimination of biological donor-to-donor variability. This manufacturing standardization is achieved through GMP-grade manufacturing, standardized processing, predefined donor and donation eligibility criteria, predefined release specifications, batch-specific quality control, quarantine release after donor re-screening, and controlled frozen storage and shipment. Donor-to-donor microbiota variability is inherent to allogeneic FMT and will be documented. Importantly, utilizing material from multiple donors ensures that any observed therapeutic effects are robust and generalizable across natural donor variation rather than potentially dependent on a single donor's specific microbiota composition. This strengthens the clinical applicability of our findings, since therapeutic FMT in routine clinical practice would similarly draw from a diverse screened donor population. We will report the number of individual donors contributing material to the study cohort and document donor and batch identity. Given that the study was not powered or specifically designed to detect donor-dependent effects, we will report donor-related characteristics where available as supplementary information; any notable associations with engraftment or outcomes will be described as exploratory observations only.

15. Quality control is performed to ensure sufficient diversity defined as a Shannon Diversity Index >2 and Richness >30 genera. Please add or explain why these numbers should be sufficient? Sufficient to achieve what? Importantly, what data are these numbers based on?

Response: We thank the reviewer for this important request for clarification. We acknowledge that the wording "to ensure sufficient diversity" may incorrectly imply that these thresholds are validated predictors of clinical efficacy. This was not our intention. The criteria Shannon Diversity Index >2 and Richness >30 genera are predefined internal release specifications in the IMPD for INTESTIFIX 001. They serve as quality control criteria to confirm a minimum level of microbial community complexity and to exclude markedly low-diversity or pathobiont-dominated donations. They are therefore "sufficient" for product release according to the predefined IMPD specifications, but they are not intended to guarantee engraftment or clinical efficacy. To our knowledge, no validated minimum donor microbiome diversity threshold has been established for successful FMT in patients with decompensated cirrhosis and ascites. We have therefore revised the manuscript to clearly describe these values as predefined minimum alpha-diversity QC/release specifications rather than as clinically validated efficacy thresholds.

16. ’Three daily doses of eight capsules’, please specify that the eight capsules are given at the same time/once daily on three consecutive days.

    Response: We thank the reviewer for this request for clarification. The reviewer is correct: the eight capsules constituting each daily dose are administered together at a single time point, once daily, on three consecutive days. To make this explicit, we have revised the relevant sentence in the Methods section to read: *"The treatment regimen consists of a single daily dose of eight capsules, taken together at one time point, on three consecutive days. This results in a total cumulative dose of 24 capsules, corresponding to approximately 40 g of original stool suspension."* We thank the reviewer for helping us describe the dosing regimen more precisely.

17. The dose was selected ’to optimize the probability of microbial engraftment and barrier restoration’, what data is this dose based on?

    Response: We thank the reviewer for this important question regarding the rationale for the selected dose. We would like to clarify the basis for our dosing decision as follows. A typical FMT dose for most indications is approximately 50 g of faecal material, although reported doses range widely from 10 to 100 g per administration (Nowak et al., 2019). Doses as low as 30 g have been shown to be effective in *Clostridioides difficile* infection (CDI) and irritable bowel syndrome, and large stool banks have documented successful outcomes with a minimum of 12.5 g for upper gastrointestinal delivery (Cammarota et al., 2019). To date, no dedicated dose-finding studies for FMT in cirrhosis have been conducted, and the available trials have used heterogeneous regimens. In the phase I capsule trial by Bajaj, Salzman, et al. (2019), 15 capsules contained 4.125 g of stool using a wet preparation without lyophilisation. The ongoing PROMISE (ISRCTN17863382) and ChiFT (NCT04932577) trials employ 5 capsules every three months and three applications of 50 g of cryopreserved, homogenised faeces, respectively. Against this background, and given the pronounced intestinal barrier dysfunction in patients with cirrhosis and ascites, a dose of 40 g was agreed upon as the optimal dose to be investigated in the TransImmune study. To improve oral tolerability and to allow close monitoring of side effects, this dose is divided into three single daily doses of eight capsules each. Extending the administration over three consecutive days is, in addition, expected to enhance microbial engraftment.

18. ’To ensure product viability, the IMP is removed from ultra-low temperature storage 10 to 30 minutes prior to administration’. Please clarify what is meant by viability?

    Response: We thank the reviewer for this comment. To avoid any ambiguity, we have rephrased the sentence and removed the term "viability." The passage now reads: “The IMP is removed from ultra-low temperature storage 10 to 30 minutes prior to administration to allow the capsules to reach a suitable temperature for intake.”

19. The first three randomised patients will be treated sequentially. Can you be sure that there will be FMT treated patients in the first three patients included? How big are the blocks in the block design?

Response: We thank the reviewer for these questions. The sentinel (sequential) treatment of the first three randomised patients is a **safety run-in** designed to detect any early, treatment-emergent SAE before parallel enrolment proceeds. Importantly, relevant risks are not confined to the FMT product itself but also arise from the administration procedure, which is common to both arms — for example, swallowing or aspiration risk associated with capsule intake, effects related to the cold temperature of the product, and general tolerability of the delivery route. Monitoring the first three patients irrespective of allocation therefore provides meaningful safety information for both FMT- and placebo-treated participants.

For this reason, and because randomisation is blinded with concealed allocation, we cannot — and deliberately do not — guarantee that any specific number of these first three patients will receive FMT rather than placebo. Guaranteeing FMT allocation among the first three patients would require unblinding or deterministic assignment, which would compromise allocation concealment and introduce selection bias. The purpose of the staggered start is thus to monitor the safety of study participation under the randomised, blinded design as a whole, not to accrue a fixed number of FMT exposures.

Regarding the block size: in accordance with standard methodology for blinded RCTs, the specific block size(s) are intentionally not disclosed and are known only to the unblinded statistician generating the allocation sequence, as knowledge of a fixed block size would allow allocation to be predicted and thereby undermine concealment.

20. In the sentence ’However, IMP administration must be permanently discontinued if .. etc.’: suggest to add ’during the treatment phase’ in the end of the sentence, as it can be misunderstood as if the treatment were going on for longer than just the three consecutive days in the beginning.

Response: We have added this specification as requested.

21. Peritoneal inflammation is mentioned as an exploratory objective. Please clarify if collection of ascites is only performed if patients need a paracentesis at follow-up and is not collected at baseline for comparison pre-FMT? In the legend for Figure 1 (Patient flow chart) it is written that Ascites fluid will be collected at scheduled visits.

Response: We thank the reviewer for this important request for clarification, and we are happy to confirm that ascitic fluid is collected as a study-mandated procedure but only when a paracentesis happens to be required during follow-up. We clarified the figure

legend as follows: “Ascitic fluid will be collected when clinically indicated diagnostic puncture/paracentesis is performed”.

22. Audits will be conducted according to the study-specific audit plan. This may include on-site visits by a member of the sponsor's quality assurance unit or an authorized independent auditor’. Whether the audits are conducted by a person working for the

Response: We have clarified the section as follows: “Audits will be conducted according to the audit plan for this study. This includes the possibility that a member of the sponsor's quality assurance unit or a person authorized by the sponsor (auditor) visits the study site on site to check the study conduct. Similarly, all study documents originating from the study site may be audited. Auditors carry out their work independently of studies. In the case of a regulatory audit (inspection), the auditor will be accompanied by a monitor or other authorized person from the sponsor. The sponsor and all participating study sites are obliged to support auditors and inspectors of competent authorities and, in this context, to grant the persons commissioned access to the original documents.“

23. it is mentioned that an independently DSMB will periodically review safety data. It is recommended to specify before start of trial how often / after how many patients included (or according to other criteria) this happens.

Response: We thank the reviewer for this recommendation. An independent Data and Safety Monitoring Board (DSMB), comprising three voting members (two clinicians and an independent biostatistician), provides continuous oversight of participant safety throughout the trial. The schedule and criteria for its scheduled safety reviews, together with its membership, operation, and procedures, are pre-specified in the DSMB Charter, in addition to ad hoc reviews triggered by any safety concern. Accordingly, we have added the following clarification to the manuscript:

"An independent Data and Safety Monitoring Board (DSMB), comprising two clinicians and an independent biostatistician, continuously monitors participant safety throughout the trial; the schedule and criteria for its safety reviews are pre-specified in the DSMB Charter, in addition to ad hoc reviews triggered by any safety concern."

Reviewer 2 comments:

24. Strengths and limitations - line 64: I would suggest adding very to statistical power is limited.

    Response: We thank the reviewer for this helpful suggestion regarding the wording of the limitation in line 64. In response to an earlier comment from the Editor, the entire 'Strengths and limitations of this study' section has been revised to focus specifically on the study methodology. In the course of this revision, we have also taken the reviewer's suggestion into account and emphasised the constrained statistical power accordingly.

25. Line 67-9: I agree with their pragmatic "real-world" approach but given 12 per group - even with randomisation there is a high risk these medications will not be balanced between groups. Of course, this does not matter for primary endpoint of safety.

    Response: We thank the reviewer for this thoughtful and astute observation. We fully agree that, with only 12 participants per group, randomisation cannot reliably ensure a balanced distribution of these concomitant medications (e.g. lactulose, proton pump inhibitors, and antibiotic prophylaxis) between the two arms, and that this imbalance may complicate the interpretation of microbiome-related changes. As the reviewer rightly notes, however, this does not compromise the primary endpoint of the trial, which is the assessment of safety and tolerability. We have retained the corresponding bullet point in the 'Strengths and limitations' section to transparently acknowledge this potential imbalance as a limitation of the exploratory, microbiome-related analyses, while emphasising that the trial is designed and powered primarily to evaluate safety.

26. Line 81: I would suggest describing this as a key mechanism as central to progression is the driver for disease - most commonly alcohol and then MASLD in decompensated cirrhosis.

    Response: We thank the reviewer for this valuable and nuanced comment. We fully agree that the underlying aetiology of cirrhosis — most commonly alcohol-related liver disease and, increasingly, MASLD — represents the primary driver of disease, and that dysfunction of the gut-liver axis is more accurately described as a key mechanism contributing to disease progression rather than as its central cause. We have revised the wording accordingly. The sentence now reads: *"While the underlying aetiology drives the development of cirrhosis, a key mechanism underlying its progression to decompensation is dysfunction of the gut-liver axis."*

27. Line 198 - is alcohol consumption permitted

    Response: We thank the reviewer for this relevant question. Alcohol consumption is not prohibited during the study, and ongoing alcohol use does not constitute an exclusion criterion. While we encourage alcohol abstinence in the sense of an aetiological cure, we

have deliberately chosen not to make abstinence a condition of participation, in order to ensure that the study population reflects the "real-world" patient as closely as possible. During the inpatient stay on our ward, alcohol consumption is prohibited under the general ward rules. To clarify this in the manuscript, we have added the following sentence to the Methods section: *"Alcohol consumption is not an exclusion criterion and is not prohibited during the outpatient phase of the study; however, abstinence is encouraged, and alcohol use is not permitted during inpatient stays in accordance with ward regulations."*

28. Line 278: It states patients will be watched swallowing the capsules - are the patients hospitalised for these 3 days or is this via mobile phone footage?
We thank the reviewer for this clarifying question. Capsule administration is directly supervised in person by study personnel and is not monitored remotely (e.g., via mobile phone footage). This is necessitated in part by the storage requirements of the IMP, which must be kept frozen in a ultra-low temperature freezer at −80 °C until administration. To make this explicit, we have revised the text as follows: *"The IMP, whether INTESTIFIX 001 or placebo capsules, will be taken under the supervision of study personnel at the study site with clear water, following a fasting period of at least two hours."* Administration on the three consecutive days therefore takes place at the study site under direct observation — either during an inpatient stay or, for outpatients, at scheduled on-site visits on each of the three days.

29. Do the team plan an ascitic tap within 48 hrs of first dosing to exclude SBP?
We thank the reviewer for this question. A dedicated ascitic tap within 48 hours of first dosing is **not** planned as a study-defined procedure. Ascitic paracentesis is instead governed by standard of care and is performed on unplanned hospitalisation, new or worsening decompensation, new organ dysfunction, or clinical suspicion of infection.In accordance with German and institutional guidelines, patients routinely undergo an ascitic tap on admission to exclude SBP, and a positive finding precludes enrolment. Where SBP is clinically suspected at or near the time of dosing, a diagnostic tap is likewise performed as part of routine care. Active infection, including SBP, is therefore reliably excluded through standard clinical assessment rather than through an additional study-specific puncture within a fixed pre-dosing window.

30. Line 283 - what time gap between treating the first 3 patients is planned to observe AEs - ?1 week

Response: We thank the reviewer for this question. To address the requested observation interval, dosing of the first three patients is staggered such that each patient is observed throughout the three-day treatment phase before the subsequent patient is dosed. If no IMP-related serious adverse reaction (SAR) is recorded during this period, recruitment of

the remaining cohort proceeds without restriction; if an IMP-related SAR occurs, the next three patients are again dosed sequentially. To clarify this in the manuscript, the wording has been revised to: *"As a specific safety precaution, the first three randomised patients will be dosed sequentially, with each patient observed throughout the three-day treatment phase before the next patient is dosed. Recruitment of the remaining cohort will proceed without restrictions if no IMP-related serious adverse reactions (SARs) are recorded during the treatment phase of these sentinel participants; if an IMP-related SAR occurs, the subsequent three patients are likewise dosed sequentially."*

VERSION 2 - REVIEW

**Reviewer**	**1**
**Name**	**Thomsen, Karen Louise**
**Affiliation**	**Aarhus University Hospital, Department of Hepatology and Gastroenterology**
**Date**	**10-Jul-2026**
**COI**	

The authors responses are elaborate and sufficient and appropriate changes to the manuscript have been made. Good luck with the study!
